# Lamotrigine for cognitive deficits associated with neurofibromatosis type 1: A phase II randomized placebo‐controlled trial

**DOI:** 10.1111/dmcn.16094

**Published:** 2024-09-28

**Authors:** Myrthe J. Ottenhoff, Sabine E. Mous, Jesminne Castricum, André B. Rietman, Rianne Oostenbrink, Thijs van der Vaart, Joke H. M. Tulen, Alba Parra, Federico J. Ramos, Eric Legius, Henriette A. Moll, Ype Elgersma, Marie‐Claire Y. de Wit, ENCORE Expertise Center for NF1, Walter Taal, Sarah van Dijk, Laura de Graaff, Yvette van Ierland, Margreethe van Vliet, Jeroen Legerstee, Pieter de Nijs, Maarten van Schijndel

**Affiliations:** ^1^ Department of Neuroscience Erasmus Medical Center Rotterdam the Netherlands; ^2^ Department of Pediatrics Erasmus Medical Center Sophia Children's Hospital Rotterdam the Netherlands; ^3^ Erasmus MC Center of Expertise for Neurodevelopmental Disorders (ENCORE) Erasmus Medical Center Rotterdam the Netherlands; ^4^ Department of Child and Adolescent Psychiatry and Psychology Erasmus Medical Center Sophia Children's Hospital Rotterdam the Netherlands; ^5^ Child Brain Center, Erasmus Medical Center Sophia Children's Hospital Rotterdam the Netherlands; ^6^ Department of Psychiatry Erasmus Medical Center Rotterdam the Netherlands; ^7^ Department of Neurology Erasmus Medical Center Rotterdam the Netherlands; ^8^ Department of Neurology Hospital Sant Joan de Déu Barcelona Spain; ^9^ Center for Human Genetics University Hospital Leuven Leuven Belgium; ^10^ Department of Human Genetics Catholic University Leuven Leuven Belgium; ^11^ Department of Clinical Genetics Erasmus Medical Center Rotterdam the Netherlands; ^12^ Department of Pediatric Neurology Erasmus MC Sophia Children's Hospital Rotterdam the Netherlands

## Abstract

**Aim:**

To find proof‐of‐principle evidence for short‐term treatment with lamotrigine to improve cognitive functioning of adolescents with neurofibromatosis type 1 (NF1).

**Method:**

This was a double‐blind, parallel‐group, randomized, placebo‐controlled clinical trial (the NF1‐EXCEL trial: Examining the Cognitive and Electrophysiological benefit of Lamotrigine in Neurofibromatosis type 1; Clinicaltrials.gov identifier NCT02256124), with the aim of enrolling 60 adolescents with NF1 aged 12 to 17  years 6 months. The short‐term study intervention was 200 mg of lamotrigine taken orally for 26 weeks. The primary outcome was performance IQ tested with the Wechsler Intelligence Scale for Children, Third Edition, complemented with secondary outcomes for visuospatial learning efficacy, visual perception, visual sustained attention, fine motor coordination, attention‐deficit/hyperactivity problems, and executive functioning.

**Results:**

We screened 402 adolescents with NF1, of whom 31 (eight females) entered the study. Complete‐case analysis showed no effect of lamotrigine on either performance IQ (−0.23, 95% CI −6.90 to 6.44) or most secondary outcomes. Visual sustained attention showed a trend towards better performance in the lamotrigine group (−0.81, 95% CI −1.67 to 0.04).

**Interpretation:**

Lamotrigine did not improve cognitive functioning in adolescents with NF1. The small treatment effects make it unlikely that a larger sample size could have changed this conclusion.

## Graphical abstract

Plain language summary: https://onlinelibrary.wiley.com/doi/10.1111/dmcn.16157

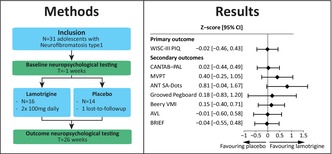

In this double‐blind, placebo‐controlled, randomized clinical trial, 31 adolescents with neurofibromatosis type 1 (NF1) were enrolled to test the effects of lamotrigine on cognitive functioning. The lamotrigine group showed no improvement in the primary outcome (performance IQ) or any secondary outcomes compared to the placebo group, suggesting lamotrigine does not enhance cognitive function in adolescents with NF1. The small treatment effects observed indicate that it is unlikely that a larger sample size would alter these findings.

This original article is commented by Payne on pages 426–427 of this issue.

**Abbreviations**
ANT‐SA Dots: ANT Sustained Attention Dots task
AVL: attention‐deficit/hyperactivity problems questionnaire (Dutch ‘Aandachtsvragenlijst’)
BRIEF: Behavior Rating Inventory of Executive Function
CANTAB‐PAL: CANTAB Paired Associates Learning task
HCN1: hyperpolarization‐activated cyclic nucleotide‐gated channel 1
NF1: Neurofibromatosis type 1

**What this paper adds**

- Lamotrigine was well tolerated in adolescents with neurofibromatosis type 1 (NF1).
- Lamotrigine did not improve performance IQ (primary outcome) in adolescents with NF1.
- Lamotrigine did not improve any secondary neurocognitive outcomes in adolescents with NF1.
- Sustained attention showed a trend towards improvement upon treatment with lamotrigine.

Neurofibromatosis type 1 (NF1) is a genetic disorder affecting up to 1:2000 births,^1^ which is caused by heterozygous pathogenic variants or deletions of the *NF1* gene. NF1 is associated with a wide range of somatic symptoms that mostly affect the skin and peripheral and central nervous system, such as (sub)cutaneous neurofibromas, optic glioma, and skinfold freckling.^2^ Additionally, cognitive and behavioural deficits are common in NF1 which have the highest impact on quality of life at a young age.^3^, ^4^ Studies consistently show that individuals with NF1 have an IQ of about 1SD below the general population.^5^ The more specific neurocognitive domains affected in NF1 are attention, working memory, visuospatial ability, executive function, and fine motor skill.^6^, ^7^ Moreover, rates of attention‐deficit/hyperactivity disorder (ADHD) and autism spectrum disorder are about 10‐fold those in the general population.^8^, ^9^, ^10^

In search of a treatment for these NF1‐related cognitive and behavioural symptoms, several clinical trials have been performed in the past decade. Most of these trials focused on using statins as a pharmacological intervention to reduce Ras activity, which is dysregulated in NF1 and considered an underlying mechanism of cognitive and behavioural problems in NF1.^11^, ^12^, ^13^ However, these trials predominantly failed to show an effect on cognitive or behavioural outcomes, with only two smaller trials showing small treatment effects.^14^, ^15^, ^16^, ^17^, ^18^ Consequently, these trials did not lead to the implementation of statins in the clinical management of cognitive and behavioural deficits in individuals with NF1.

Meanwhile, our understanding of the underlying mechanisms of cognitive and behavioural problems in NF1 has broadened. In mice, loss‐of‐function of neurofibromin results in a higher activity of hippocampal inhibitory interneurons, through reduced hyperpolarization‐activated cyclic nucleotide‐gated channel 1 (HCN1)‐induced hyperpolarization. Interestingly, lamotrigine, a widely used antiseizure and mood‐stabilizing drug, is a known agonist of HCN1. Lamotrigine administration in *Nf1* mutant mice was subsequently able to restore HCN1 function along with alleviating their memory and learning deficits.^19^ More recently, it was shown that attenuated HCN function in peripheral neurons of *Nf1* mutant mice resulted in hyperexcitability and increased tumour progression, which could be ameliorated upon treatment with lamotrigine.^20^ These findings suggest that treatment with lamotrigine could be beneficial for individuals with NF1, even beyond treating the cognitive deficits.

The primary objective of this trial was to find proof‐of‐principle for the effect of a short‐term lamotrigine intervention (26 weeks) on cognitive functioning in adolescents with NF1. Secondary objectives of this trial were to evaluate the safety of lamotrigine in NF1 and the effect on specific subdomains of cognitive functioning.

## METHOD

### Study design

The NF1‐EXCEL trial is a phase II double‐blind, parallel‐group, randomized, placebo‐controlled clinical trial, performed at the ENCORE expertise centre in the Erasmus Medical Center (Rotterdam, the Netherlands). Originally, the trial was designed as a multicentre trial; however, owing to COVID‐19 restrictions and (related) logistical issues, it was impossible to recruit participants at the collaborating centres (University Hospital Leuven in Leuven, Belgium, and the Hospital Sant Joan de Déu in Barcelona, Spain). The trial is registered at clinicaltrials.gov (https://clinicaltrials.gov/ct2/show/NCT02256124).

The sample size was calculated to be 46 participants in total using G*power software (version 3.1; Düsseldorf, Germany).^21^ As we planned to use an analysis of covariance (ANCOVA) for the outcome analysis, sample size calculation was corrected on the basis of the test–retest reliability (*ρ*) of the primary outcome as previously described.^22^, ^23^ This correction entails multiplication of the sample size for a two‐sample *t*‐test by a factor of (1 − *ρ* ^2^). The parameters to calculate the sample size were an effect size of 0.5 on the primary outcome (which equates to a 7.5 IQ point treatment effect), a power of 80%, a two‐sided *α* of 0.05, and a *ρ* of 0.8. A *ρ* of 0.8 for performance IQ (primary outcome) was a conservative estimate based on previous studies in children with NF1.^24^ We decided to increase the intended sample size from 46 to 60 participants to accommodate expected non‐compliance, those lost to follow‐up, and possible unbalanced treatment arms owing to stratification per centre.

### Participants

We recruited participants mainly through the ENCORE NF1 outpatient clinic. Additionally, we asked other national NF1 outpatient clinics to distribute our study invitation material among potentially eligible individuals. For eligibility screening we used the following inclusion criteria: aged 12 to 17 years 6 months at inclusion; a clinical NF1 diagnosis that was genetically confirmed; and oral and written informed consent. Genetic counselling and testing for changes in the *NF1* gene are part of routine clinical care and were done independently of this trial. Exclusion criteria were segmental NF1; severely impaired vision or hearing; use of medication known to influence lamotrigine blood levels (phenytoïn, carbamazepine, phenobarbital, primidon, rifampicine, atazanavir/ritonavir, lopinavir/ritonavir, oxcarbazepine, topiramate, hormonal oral contraceptive pill, or valproic acid) during the previous 3 months; psychoactive medication other than methylphenidate (for methylphenidate use, stable dosage for at least 3 months, both immediate and extended release allowed) because methylphenidate was estimated to be the most commonly used psychoactive medication in our target population on the basis of ADHD prevalence^9^, ^10^ and ADHD management guidelines;^25^ previous use of lamotrigine; previous allergic reactions to antiseizure medication; current or previous epilepsy; suicidal thoughts or behaviour; renal and/or liver insufficiency; pregnancy; symptomatic brain pathology.

Informed oral and written consent from parents and assent from adolescents themselves was obtained. National and Erasmus Medical Center institutional review boards approved the protocol (registration number MEC‐2013‐460), of which the last version is included as Appendix S1. Additionally, the statistical analysis plan (Appendix S2) includes a version history of the study protocol. The trial was conducted in agreement with the Declaration of Helsinki and Good Clinical Practice guidelines.

### Randomization and blinding

Participants were randomly assigned by the local hospital pharmacist to lamotrigine or placebo in a 1:1 ratio. A computer‐generated permuted‐block randomization list was generated (block size of six participants), which was provided by the Department of Biostatistics of the Erasmus Medical Center, with medication numbers in the order of enrolment. The randomization list was kept at the Erasmus Medical Center trial pharmacy, who also performed intervention assignment. Enrolment of participants was performed by the investigators. All investigators, participants, and their parents were blinded for treatment allocation, until the database was locked and the statistical analysis plan (Appendix S2) was finalized and signed by the research team.

### Intervention

Intervention consisted of lamotrigine in a dose of 200 mg/day in two doses of 100 mg (morning and evening) taken orally or a matching placebo over a short‐term intervention period of 26 weeks. The target dosage was reached by gradually increasing the dosage during the initial 8 weeks, followed by an 18‐week target dose phase, before finally being tapered in 2 weeks. More specifically, in weeks 1 and 2, participants received a dosage of 25 mg/day; in weeks 3 and 4, 50 mg/day; in weeks 5 and 6, 100 mg/day; in weeks 7 and 8, 150 mg/day; and finally in weeks 9 to 26, 200 mg/day. During weeks 27 and 28, tapering consisted of lowering the dose to 100 mg/day, before completely discontinuing lamotrigine in week 29. During the study period, dose reductions were accepted in cases of dose‐related side effects that would cause participants to discontinue the stud.

Lamotrigine was administered in the form of dispersible tablets. We used 25 mg tablets during the dose escalation phase and 100 mg tablets during the maintenance phase. Lamotrigine was produced by Teva (Haarlem, the Netherlands) and repackaged and labelled by the trial pharmacy of the Erasmus Medical Center (Rotterdam, the Netherlands) into opaque white plastic bottles. Placebo tablets were produced by the pharmacy Haagse Ziekenhuizen (The Hague, the Netherlands) for this study specifically to match colour, shape, imprint, size, fill material, and dispersibility, and were packaged and labelled identically to lamotrigine tablets by the trial pharmacy of the Erasmus Medical Center.

### Outcomes

A broad range of validated neuropsychological tests and questionnaires were used, which are expected to be sensitive to the cognitive deficits in adolescents with NF1. These tests and questionnaires were selected on the basis of previous research and clinical relevance.^24^ We assessed outcome measures at baseline (*T* = −1 week, 1 week before start of medication), after 10 weeks of medication use (*T* = 10 weeks), at the end of the study period (at *T* = 26 weeks), and after a period of 26 weeks without medication (*T* = 52 weeks).

The primary outcome measure was performance IQ at *T* = 26 weeks, as measured with the Wechsler Intelligence Scale for Children, Third Edition.^26^ For children exceeding the Wechsler Intelligence Scale for Children, Third Edition age limit (16 years 11 months) during one or both assessments, a linear extrapolation of normative values was performed to allow the calculation of age‐appropriate standard scores. Secondary outcome measures included several neuropsychological tests and questionnaires at *T* = 26 weeks: visuospatial learning efficacy (as measured with the CANTAB Paired Associates Learning task [CANTAB‐PAL]^27^); visual perception (Motor‐Free Visual Perception Test, Third Edition^28^); visual sustained attention (ANT Sustained Attention Dots task [ANT‐SA Dots]^29^); fine motor coordination (Grooved Pegboard Test^30^) and the motor coordination subtest from the Beery‐Buktenica Developmental Test of Visual Motor Integration, Sixth Edition^31^); parent/caregiver‐rated attention‐deficit/hyperactivity problems (using the Dutch questionnaire ‘Aandachtsvragenlijst’ [AVL]^32^); and parent/caregiver‐rated executive functioning (Behavior Rating Inventory of Executive Function [BRIEF] questionnaire^33^).

Additionally, the AVL was measured at *T* = 10 weeks (during the intervention period) and both the AVL and BRIEF were measured at *T* = 52 weeks (26 weeks after the end of the intervention period), although these time points are not reported in this paper. The rationale for including these measurements at these specific time points in the study was to enable exploratory analysis of the stability of the hypothesized treatment effect; they were not predefined as actual efficacy outcomes for this trial. We deemed it inappropriate to include such exploratory analysis in the statistical analysis plan, owing to the low inclusion number (see Results).

All instruments were developed for adolescents and were written or presented in the Dutch language. For most outcome measures, age‐standardized scores were used. The mean average performance IQ for the general population is 100 (SD 15), with higher performance IQ scores indicating higher performance intelligence. For the CANTAB‐PAL, Motor‐Free Visual Perception Test, and Beery‐Buktenica Developmental Test of Visual Motor Integration, Sixth Edition, data were represented as z‐scores, with higher scores indicating better performance. For the ANT‐SA Dots, data were represented as a z‐score, where a higher score indicates worse performance. Data for the Grooved Pegboard Test were represented as a raw score, with a higher score indicating worse performance. Finally, for the AVL and BRIEF questionnaires, data were represented as z and *T* scores respectively, both indicating more problems with higher scores. All assessments were performed by a licensed psychologist or trainee (supervised by the psychologist), and assessments during the different time points were done by the same person.

Adverse events and study compliance were monitored by telephone contacts at *T* = 4, *T* = 8, and *T* = 14 weeks, and by a home visit at *T* = 18 weeks. Adverse events were classified according to World Health Organization adverse reaction terminology and graded in line with the National Cancer Institute Common Terminology Criteria for Adverse Events. Blood was drawn at *T* = 10 weeks to assess liver function and blood count as a safety measure. Haematological or biochemical values that were out of range were considered adverse events.

Compliance is defined as the number of tablets consumed out of the number of tablets prescribed, monitored by counting returned tablets. Adherence is defined as being on study medication at the time of follow‐up outcome assessment and a compliance greater than 80%, as assessed by the percentage of consumed tablets out of the expected number of consumed tablets.

Lamotrigine serum levels were drawn at three visits during the target dose phase (*T* = 10 weeks, 18 weeks, and 26 weeks), during which each participant had a blood sample drawn timed at any of three time points relative to their last lamotrigine dose intake (a *T*_max_ level aimed at around 3 hours after intake, an intermediate level aimed around 6 hours after intake, and a trough level aimed as close to the evening dose intake as possible). Lamotrigine blood levels were analysed by a homogenous enzyme immunoassay (ARK Diagnostics, Fremont, CA, USA), using an automated analyser (Abbott Architect C4000, Chicago, IL, USA). The range of quantification of the method was 0.85 to 40 mg/L.

For data capture and management, we used OpenClinica Community 3.12.2 software (Waltham, MA, USA).

### Statistical analysis

We performed a complete‐case analysis, meaning we analysed all participants with primary outcome data, regardless of the level of compliance. We analysed primary and secondary outcomes using an ANCOVA model: a linear model assessing the effect of treatment group on the outcome at *T* = 26 weeks, controlled for the baseline measurement at *T* = −1 week.

We checked the normality assumption of the residuals for these models by inspecting Q–Q plots. These plots indicated a likely violation of the normality assumption for the Grooved Pegboard Test, AVL, and BRIEF outcomes. We then explored whether square‐root or logarithmic transformations improved the normality of the residuals in these Q–Q plots. This approach only showed improvement for the AVL outcome after a logarithmic transformation. For the Grooved Pegboard Test and BRIEF outcomes, where transformations did not sufficiently improve normality, we conducted an additional non‐parametric analysis. This analysis was achieved by first ranking the outcome (both at *T* = 26 weeks and at the baseline measurement *T* = −1 week), before conducting the ANCOVA model as described above. The non‐parametric models for the Grooved Pegboard Test and BRIEF, as well as the logarithmic‐transformed model for AVL, did not alter the significance level of the treatment effect. Therefore, for ease of interpretation, we report the results from the parametric linear models in the main Results section. The results from the alternative models are provided in Table S1.

The constant variance assumption was checked by inspecting plots of the fitted values against the square‐root‐transformed standardized residuals. This assessment revealed a potential violation for this assumption for the CANTAB‐PAL, Motor‐Free Visual Perception Test, and ANT‐SA Dots outcomes. To address this heteroscedasticity, we used robust standard errors using the heteroskedasticity‐consistent 1 (HC1) estimator, which is a correction based on degrees of freedom that is more suitable for small samples.^34^

The significance level was set at *p* < 0.05, without adjusting for multiple testing. The statistical analysis plan (Appendix S2) and syntax were developed by the research team under supervision of an independent statistician. They were finalized and subsequently approved by the independent statistician and the core research team in November 2021, before unblinding the study in April 2022. After unblinding, the statistical analyses were performed by the research team as specified before unblinding in the statistical analysis plan, with the exception of the non‐parametric Grooved Pegboard Test and BRIEF models. All statistical analyses were performed using R programming language (version 4.0.3; Vienna, Austria)^35^ in RStudio (version 1.3.1093; Boston, MA, USA).^36^

## RESULTS

We screened 402 adolescents, of whom 333 were eligible. From this population, we obtained informed consent from 31 adolescents and their parents between 4th October 2014 and 30th April 2020 (Figure S1). Although the sample size for the study was originally calculated to be 60 participants (30 participants lamotrigine and 30 control), it was decided to stop inclusion for the NF1‐EXCEL study prematurely on 30th April 2020. This premature stop to the study was necessitated by several factors, including a slower inclusion rate than anticipated, COVID‐19 restrictions on inclusion and follow‐up, and logistical issues concerning the discontinuation of medication production.

The 31 participants were randomly assigned to treatment with lamotrigine (*n* = 16) or placebo (*n* = 15). One participant in the placebo group discontinued study medication during follow‐up owing to side effects and did not complete the end visit at *T* = 26 weeks. This participant, therefore, did not enter the analysis. Compliance was high in both the lamotrigine (median 95%, IQR 77–97%) and placebo (median 97%, IQR 88–98%) groups. The number of adherent participants was 11 in the lamotrigine group and 11 in the placebo group. All non‐adherent participants remained in the analysis population (Figure S1).

We analysed lamotrigine trough blood levels from all participants in the lamotrigine group, which were taken at the *T* = 18 weeks control visit as close to the evening dose as possible. The median time since last intake was 9.54 hours (range 6.80–11.00). Lamotrigine trough samples had a median blood serum level of 4.68 mg/L (range 1.54–7.36), with only one participant having a level below the therapeutic range of 3 to 14 mg/L.^37^, ^38^ We additionally analysed lamotrigine blood samples drawn around the *T*_max_ (3 hours after last intake) and an intermediate level (6 hours after last intake), for which the results are summarized in Table S2.

Baseline demographic and disease characteristics were mostly similar between groups (Table 1), although the placebo group seemed to have more children who attended special education and a higher rate of autism spectrum disorder. The overall median age at inclusion was 14 years 5 months (IQR 12 years 11 months–15 years 2 months). Eight participants were female. Familial inheritance of NF1 was observed in seven of the participants. The mean Full‐scale IQ was 87.4 (SD 15.9), which is comparable to previously reported results.^5^

**TABLE 1:** Baseline demographics and disease characteristics.

	Lamotrigine (*n* = 16)	Placebo (*n* = 14)
Age, years:months	14:0 (12:11–15:1)	14:10 (13:2–16:1)
Females	5 (31.3%)	3 (21.4%)
Full‐scale IQ^a^	87.6 (18.1)	87.1 (13.7)
Verbal IQ^a^	90.1 (17.4)	86.8 (18.0)
Performance IQ^a^	88.0 (20.1)	89.4 (10.6)
Intellectual disability^b^	3 (18.8%)	2 (14.3%)
NF1 disease severity^c^		
Minimal	5 (31.3%)	4 (28.6%)
Mild	4 (25.0%)	6 (42.9%)
Moderate	4 (25.0%)	4 (28.6%)
Severe	3 (18.8%)	0 (0.0%)
Mutation type		
Missense	2 (12.5%)	4 (28.6%)
Frameshift	4 (25.0%)	4 (28.6%)
Splicing	4 (25.0%)	3 (21.4%)
Nonsense	4 (25.0%)	1 (7.1%)
Microdeletion	2 (12.5%)	1 (7.1%)
Familial inheritance	4 (25.0%)	3 (21.4%)
Education type		
Regular	5 (31.3%)	2 (14.3%)
Remedial teaching	5 (31.3%)	3 (21.4%)
Special education	6 (37.5%)	9 (64.3%)
Maternal education^d^		
Lower	11 (68.8%)	9 (64.3%)
Higher	5 (31.3%)	5 (35.7%)
Attention‐deficit/hyperactivity disorder		
Positive diagnosis	7 (43.8%)	6 (42.9%)
Unknown	1 (6.7%)	1 (7.1%)
Autism spectrum disorder		
Positive diagnosis	1 (6.3%)	5 (35.7%)
Unknown	2 (12.5%)	1 (7.1%)
Methylphenidate use	5 (31.3%)	6 (42.9%)

Data are median (25th–75th centiles), mean (SD), or number (percentage).

^a^ Measured with the Wechsler Intelligence Scale for Children, Third Edition.

^b^ Intellectual disability is defined as a Full‐scale IQ < 70.

^c^ Measured with the Riccardi scale that was modified to exclude cognitive symptoms.^57^

^d^ Higher maternal education is defined as higher vocational education, bachelor's, master's, or doctoral degree.

**FIGURE 1:**
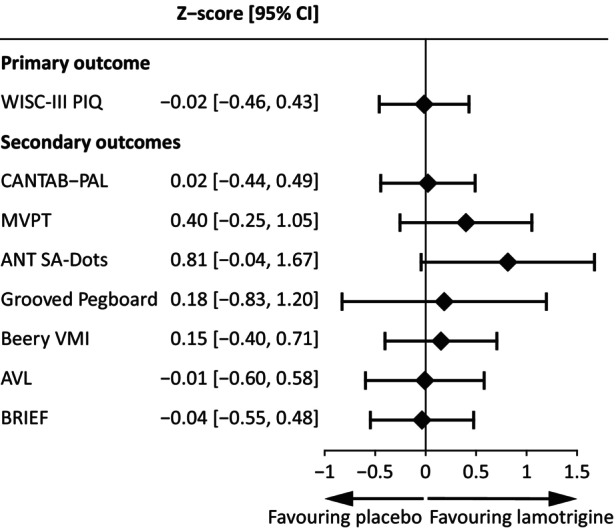
Standardized treatment effects. The effect of treatment with lamotrigine on primary and secondary outcome measures, adjusted for baseline performance. Higher z‐scores favouring lamotrigine. Abbreviations: ANT‐SA Dots, ANT Sustained Attention Dots task; AVL, attention‐deficit/hyperactivity problems questionnaire (Dutch ‘Aandachtsvragenlijst’); Beery‐VMI, Beery‐Buktenica Developmental Test of Visual Motor Integration, Sixth Edition; BRIEF, Behavior Rating Inventory of Executive Function; CANTAB‐PAL, CANTAB Paired Associates Learning task; CI, confidence interval; MVPT, Motor‐Free Visual Perception Test; PIQ, performance IQ; WISC‐III, Wechsler Intelligence Scale for Children, Third Edition.

**FIGURE 2:**
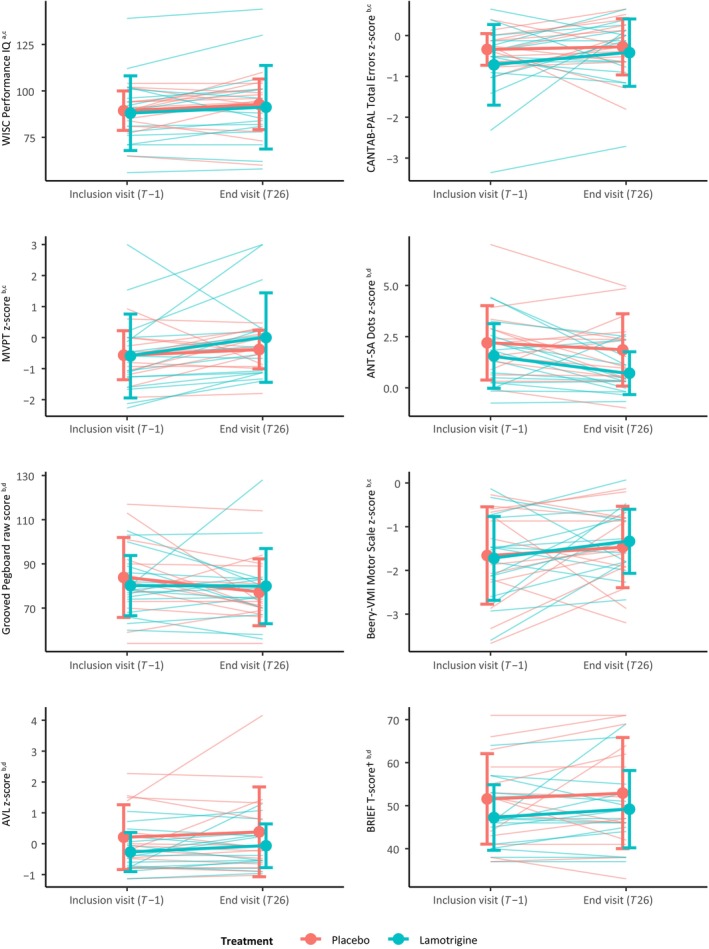
Treatment effects per outcome and treatment group. Dots and thick lines, group means; whiskers, the standard error; thin lines, individual treatment effects. ^a^Primary outcome. ^b^Secondary outcome. ^c^A higher score indicates higher performance. ^d^A lower score indicates higher performance. Abbreviations: ANT‐SA Dots, ANT Sustained Attention Dots task; AVL, attention‐deficit/hyperactivity problems questionnaire (Dutch ‘Aandachtsvragenlijst’); Beery‐VMI, Beery‐Buktenica Developmental Test of Visual Motor Integration, Sixth Edition; BRIEF, Behavior Rating Inventory of Executive Function; CANTAB‐PAL, CANTAB Paired Associates Learning task; MVPT, Motor‐Free Visual Perception Test; WISC‐III, Wechsler Intelligence Scale for Children, Third Edition.

At the end visit (*T* = 26 weeks), treatment with lamotrigine did not have a significant effect on the primary outcome measure, performance IQ (treatment effect −0.23, 95% CI –6.90 to 6.44, *p* = 0.95), when adjusted for baseline performance. Additionally, on none of the secondary outcomes were significant treatment effects found (Table 2 and Figures 1 and 2). A trend was observed for the sustained attention task (ANT‐SA Dots) (treatment effect −0.81, 95% CI −1.67 to 0.04, *p* = 0.07).

**TABLE 2:** Primary and secondary outcomes.

	Visit (weeks)	Lamotrigine (*n* = 16)	Placebo (*n* = 14)	Treatment effect (95% CI)	*p*
**Primary outcome**					
WISC‐III performance IQ^a^				−0.23 (−6.90 to 6.44)	0.95
	Inclusion (*T* − 1)	88 (20.1)	89.4 (10.6)		
	End (*T* = 26)	91.2 (22.5)	92.8 (13.6)		
**Secondary outcomes**					
CANTAB‐PAL total errors z‐score^a^				0.02 (−0.44 to 0.49)^b^	0.92^b^
	Inclusion (*T* − 1)	−0.7 (1.0)	−0.3 (0.4)		
	End (*T* = 26)	−0.4 (0.8)	−0.3 (0.7)		
MVPT z‐score^a^				0.4 (−0.25 to 1.05)^b^	0.24^b^
	Inclusion (*T* − 1)	−0.6 (1.4)	−0.6 (0.8)		
	End (*T* = 26)	0 (1.4)	−0.4 (0.6)		
ANT‐SA Dots z‐score^c^				−0.81 (−1.67 to 0.04)^b^	0.07^b^
	Inclusion (*T* − 1)	1.6 (1.6)	2.2 (1.8)		
	End (*T* = 26)	0.7 (1.1)	1.9 (1.8)		
Grooved Pegboard Test raw score^c^, ^d^				4.82 (−5.17 to 14.82)	0.35
	Inclusion (*T* − 1)	80.2 (13.6)	83.9 (18.1)		
	End (*T* = 26)	79.9 (17.0)	77.1 (15.1)		
Beery‐VMI motor coordination z‐score^a^				0.15 (−0.40 to 0.71)	0.60
	Inclusion (*T* − 1)	−1.7 (1.0)	−1.7 (1.1)		
	End (*T* = 26)	−1.3 (0.7)	−1.5 (0.9)		
AVL z‐score^c^, ^e^				0.01 (−0.58 to 0.60)	0.98
	Inclusion (*T* − 1)	−0.3 (0.6)	0.2 (1.1)		
	End (*T* = 26)	−0.1 (0.7)	0.4 (1.5)		
BRIEF *T* score^c^, ^d^				0.36 (−4.76 to 5.48)	0.89
	Inclusion (*T* − 1)	47.3 (7.6)	51.6 (10.5)		
	End (*T* = 26)	49.2 (9.0)	52.9 (12.9)		

Mean (SD) of the primary outcome and the secondary outcomes by treatment group at inclusion and end visit.

Abbreviations: ANT‐SA Dots, ANT Sustained Attention Dots task; AVL, attention‐deficit/hyperactivity problems questionnaire (Dutch ‘Aandachtsvragenlijst’); Beery‐VMI, Beery‐Buktenica Developmental Test of Visual Motor Integration, Sixth Edition; BRIEF, Behavior Rating Inventory of Executive Function; CANTAB‐PAL, CANTAB Paired Associates Learning task; CI, confidence interval; MVPT, Motor‐Free Visual Perception Test; WISC‐III, Wechsler Intelligence Scale for Children, Third Edition.

^a^ A higher score indicates higher performance.

^b^ The confidence intervals and *p*‐values of these treatment effects were subject to a robust standard errors correction to address heteroscedasticity in the linear model. The alternative model without robust standard errors is reported in Table S1.

^c^ A lower score indicates higher performance.

^d^ This model probably violates the normality of residuals assumption, which did not improve using either a square‐root or logarithmic transformation of the outcome. Therefore, these outcomes were reassessed using a non‐parametric model (Table S1).

^e^ This model probably violates the normality of residuals assumption, which improved when reassessing this model after logarithmic transformation of the outcome that is reported in Table S1.

Most adverse events were classified as mild or moderate and the frequency of adverse events was similar between groups (Table 3). Central and peripheral nervous system disorders where the most common category of adverse events reported in both treatment groups, out of which most events were headache‐related complaints in both the lamotrigine (83%) and placebo (89%) groups. There were two serious adverse events reported in total. One occurred in the lamotrigine group, consisting of two simultaneously occurring grade 3 adverse events of ataxia and aphasia. The participant involved needed hospitalization for 1 day, after which the adverse events spontaneously recovered without sequelae. The second serious adverse event occurred in the placebo group, consisting of a complex partial seizure for which hospitalization was required. The participant involved was unblinded for the research team to ensure optimal treatment of the seizures, after which oxcarbazepine was started and the seizures did not return for the duration of the follow‐up.

**TABLE 3:** Adverse events.

	Lamotrigine (*n* = 16)			Placebo (*n* = 14)		
WHO category	Grade 1	Grade 2	Grade 3	Grade 1	Grade 2	Grade 3
Autonomic nervous system disorders	0	1 (1)	0	0	0	0
Body as a whole: general disorders	15 (8)	0	0	22 (10)	2 (2)	0
Central and peripheral nervous system disorders	30 (12)	2 (1)	2 (1)	27 (10)	4 (3)	1 (1)
Gastro‐intestinal system disorders	25 (8)	3 (2)	0	32 (10)	2 (1)	0
Heart rate and rhythm disorders	1 (1)	0	0	0	0	0
Musculo‐skeletal system disorders	1 (1)	0	0	0	0	0
Platelet, bleeding, and clotting disorders	1 (1)	0	0	1 (1)	0	0
Psychiatric disorders	12 (8)	2 (1)	0	8 (3)	5 (4)	0
Red blood cell disorders	2 (2)	0	0	2 (2)	0	0
Resistance mechanism disorders	1 (1)	0	0	1 (1)	0	0
Respiratory system disorders	5 (4)	0	0	15 (9)	0	0
Skin and appendages disorders	8 (6)	1 (1)	0	4 (4)	1 (1)	0
Vascular (extracardiac) disorders	0	1 (1)	0	0	0	0
Vision disorders	3 (3)	0	0	0	0	0
White cell and reticuloendothelial system disorders	4 (4)	0	0	0	0	0

Number of adverse events with number of involved participants between brackets. ‘WHO category’ is the adverse event categories according to the system–organ classes of the World Health Organization adverse reaction terminology. System–organ classes are rated in different grades according to severity: grade 1 is mild, no intervention needed; grade 2 is moderate, minimal local or non‐invasive intervention needed; grade 3 is severe, hospitalization indicated. Grade 4 (life‐threatening consequence) and grade 5 (death related to adverse event) did not occur in this study.

Together with the unblinding of the above‐described participant, there were a few protocol deviations. There were three out‐of‐window visits, considering the outcome visit at *T* = 26 weeks: two participants in the lamotrigine group who had their outcome measurement scheduled less than 1 week before 26 weeks of treatment (at 23 weeks and 24 weeks) and one participant in the placebo group had their outcome visit scheduled more than 2 weeks after 26 weeks (at 29 weeks). Additionally, dose reductions due to side effects were necessary for two participants in the lamotrigine group and two participants in the placebo group, in addition to one participant in the placebo group discontinuing the study completely owing to side effects (see Method and Figure S1).

## DISCUSSION

In this paper, we present the outcomes of the first double‐blind, parallel‐group, randomized, placebo‐controlled clinical trial studying the efficacy of a short‐term lamotrigine intervention on cognitive functioning in adolescents with NF1. Our results showed that treatment with lamotrigine for 26 weeks had no effect on the primary outcome, performance IQ. Moreover, we found no effect on most of the secondary outcomes: visuospatial learning efficacy, visual perception, fine motor coordination, attention‐deficit/hyperactivity problems, or executive functioning. Only one secondary outcome, visual sustained attention, showed a trend towards better performance in adolescents with NF1 who were treated with lamotrigine. As we had seven secondary neurocognitive outcomes in total and did not correct for multiple testing, we would certainly not suggest this to be a true treatment effect.

Considering the trend on the sustained attention outcome, it is interesting that lamotrigine has been suggested to have moderately positive effects on attention in adults without NF1.^39^ In children and adolescents with epilepsy or mood disorders similar trends are observed, although these effects might also be indirect and attributable to seizure control or to the mood‐stabilizing properties of lamotrigine.^40^ If we are seeing a true treatment effect on visual sustained attention, it could therefore be a general effect and it is unknown whether this is achieved in a NF1‐dependant manner.

It is important to discuss what factors could have contributed to the null results findings in the current study. A key issue that needs to be addressed is the low sample size. Regrettably, only 31 of the intended 60 (and minimally necessary 46 according to the power calculation) participants could be included owing to (COVID‐19‐related) logistical issues, leading to a potentially underpowered study. A major contributor to the low inclusion rate was a lower‐than‐expected consent rate of roughly only 10% of invited adolescents, with adolescents mainly declining participation citing that the study would pose too high a burden on daily life. To increase inclusions, we sought to expand the study to other European centres for neurofibromatosis that were enthusiastic to participate. Unfortunately, we were unable to get the study running in other centres mainly because of the COVID‐19 pandemic.

However, we can conclude that the effect sizes in our study were very small, such that reaching 46 participants is highly unlikely to change our conclusions. In particular, the effect size of the primary outcome (performance IQ) was close to zero with the confidence interval excluding the effect size used for sample size calculation (0.5SD), while in clinical trial interim analyses assuming the upper boundary of an interim confidence interval as the future treatment effect is considered optimistic.^41^ We did not further explore the level of futility in the form of a conditional power calculation, as there were no prespecified futility thresholds determined for interim analysis at the start of the study. In addition, we achieved high medication compliance and a low attrition rate, suggesting that these factors did not negatively impact our results. Together, this makes us confident that inclusion of more participants would not have led to a positive result in this study.

The lamotrigine intervention dose of 200 mg/day was chosen for several reasons. First, it is similar to the human equivalent dose of the lamotrigine intervention in the preclinical assessments. The bioavailability of peritoneal injection in mice (100%) performed in the preclinical study is similar to oral use in humans (98%). Additionally, the human equivalent dose of the dosage administered to mice in the study by Omrani et al. would maximally be 145 mg/day (see section 7.4 of the Study Protocol, Appendix S1),^19^ which is below the 200 mg/day dose in the current study. The study dose is within the bounds of a typical dose for the treatment of epilepsy in adolescents,^42^ with the antiseizure mechanism of lamotrigine being similar to that responsible for improved memory and learning in *Nf1* mutant mice.^43^ Altogether, it is unlikely that the study dose was too low for adolescents with NF1 to reach effectiveness.

The length of treatment needed to improve neurocognitive function, particularly IQ, in NF1 is not known. The intervention period of 26 weeks used in this study is relatively brief compared with similar trials with IQ as the primary outcome. Such trials in NF1 and similar groups of patients with genetic neurodevelopmental challenges have probed interventions up to 1 year in duration.^15^, ^44^ However, considering the current study was a proof‐of‐principle study without clinical precedent in individuals with NF1, we felt that a study follow‐up of 1 year or more would impose too high a burden on the study's participants. Furthermore, we included secondary neurocognitive outcomes that were anticipated to show more immediate improvements following intervention, particularly the sustained attention (ANT‐SA Dots) and working memory (CANTAB‐PAL) tasks. Given the minimal effect sizes on both the primary and secondary outcomes, it is improbable that a longer intervention period would alter the results. We, therefore, do not deem it ethical to submit a paediatric population to such a prolonged lamotrigine exposure, given the risk of side effects.

The cognitive and behavioural profile, as well as other disease characteristics, of the study group was representative of the general NF1 population at baseline. Additionally, these characteristics were largely similar between treatment groups, although the placebo group had a higher rate of autism spectrum disorder and more adolescents attending special education. It is, however, unlikely that these group differences explain the lack of significant treatment effects. We would expect that children with more developmental disabilities, such as in the placebo group, would show lower scores on our measures of interest, potentially increasing treatment effects.

Concurrent methylphenidate was taken by 11 out of 30 participants analysed. Methylphenidate is a dopamine and norepinephrine reuptake inhibitor that is often prescribed to improve attention function in ADHD. Dysregulated dopamine signalling has been linked to behavioural deficits in several preclinical studies,^45^, ^46^, ^47^ as well as a correlation between methylphenidate use and improved cognitive function in individuals with NF1.^48^ Additionally, a trial is currently underway to investigate the impact of methylphenidate on cognitive function in children and adolescents with NF1, extending beyond attention function alone.^49^ In our study, the distribution of participants using methylphenidate was balanced between the placebo and lamotrigine groups. However, it is important to acknowledge that a potential positive effect of methylphenidate on neurocognitive performance may have partly masked the treatment effects of lamotrigine. It is worth noting that sustained attention, which is expected to be particularly susceptible to such masking effects, showed the largest effect size among all the neuropsychological outcomes.

The age of the study participants was somewhat higher than in previous clinical trials aiming to improve neurocognitive deficits in NF1, which have typically included participants 8 years and older.^14^, ^15^, ^17^ Given that cognition is a developmental construct, it cannot be ruled out that lamotrigine could have an effect in a younger study population. However, the primary reason for selecting an adolescent study population is to adhere to the internationally accepted ethical principles considering medical research in children, which state that such research is permissible if the research is directly beneficial to the particular patient group, that results cannot be obtained in adults, and that it is preferable if children are able to assent in addition to parental/caregiver consent.^50^ Legally, children younger than 12 years of age are able to assent to clinical trial participation. Additionally, adolescents with NF1 show a continued need for educational support,^51^ which means targeting an age group younger than 12 years ensured that the study was ethically sound and responsive to the needs of the participants.

Moreover, preclinical study found that lamotrigine was effective in adult mice.^19^ This suggests that in mice the lamotrigine effect is not limited to certain developmental windows, further supporting the selection of an adolescent study population over a younger population for this proof‐of‐principle trial. Additional reasons for selecting a study population aged younger than 12 years were of a practical nature. Specifically, lamotrigine dosing regimens for those younger than 12 years are weight adjusted, making blinded dosing unfeasible for this investigator‐initiated trial. These reasons combined with the ethical rationale motivated us to select participants aged 12 years and older.

Selection bias was very unlikely to have impacted the current study for the following reasons: (1) we used rigorous randomization practices, resulting in similar demographics and NF1 characteristics in both groups; (2) groups were highly similar in adherence and adverse event numbers; and (3) lost to follow‐up was limited to one participant in the placebo group. This indicates that selection bias was minimal. However, we should consider that selection bias could have been introduced owing to adolescents with more NF1‐related symptoms, including cognitive problems, being more likely to be in active follow‐up or being more motivated to participate in the study. We see, however, that the mean Full‐scale IQ in our study is very similar to that found in NF1 cohort studies.^5^, ^52^ Additionally, most of our study population had a minimal to mild disease severity, similar to those in other clinical NF1 studies.^15^, ^18^ Both factors suggest that our sample was representative of the general adolescent population with NF1.

The negative findings in this study add to the existing negative results of nearly all trials attempting to translate preclinical studies into treatments for NF1‐related cognitive and behavioural deficits.^14^, ^15^, ^16^, ^17^, ^18^ These preclinical studies are mainly performed in *Nf1* mouse models, which are considered a valid disease model, as they show learning and memory deficits along with distinct visuospatial difficulties, mirroring the NF1 cognitive phenotype. The preclinical results all converge onto the same neurophysiological mechanism that links learning and memory deficits in *Nf1* mice with decreased plasticity that originates from an increased firing of inhibitory interneurons.^11^, ^13^, ^19^ Importantly, the relation between increased inhibition and cognitive deficits is also observed in individuals with NF1.^53^ Therefore, the common rationale of these trials, including the current one, is that when this neurophysiological mechanism is corrected, the cognitive function in individuals with NF1 will improve. However, the repeated null results findings in NF1 clinical trials emphasizes the need to scrutinize certain challenges in translating treatments from mouse model studies to human clinical trials.

One translational challenge for NF1 trials is that preclinical studies have linked various molecular mechanisms to the neurophysiological mechanism described above. While the current study assumes that the attenuation of the HCN1 is the driving mechanism of the increased inhibitory interneuron firing frequency, other negative trials targeted the dysregulation of the Ras signalling pathway. Additionally, dopamine signalling is also demonstrated to be involved.^54^ It remains uncertain what the relation between these molecular mechanisms is, and thus whether these mechanisms have the ability to block treatment effects in individuals with NF1.

Another translational challenge is choosing relevant outcome measures. Some argue that outcomes in NF1 clinical trials are not similar enough to outcomes in preclinical studies, promoting, for example, visuospatial outcomes that are very close to the Morris Water Maze task often used in preclinical studies with mice.^55^ However, for a trial to have the potential to change clinical management of individuals with NF1, outcomes should both capture the neurocognitive profile of individuals with NF1 and be related to their daily functioning,^24^ such as the primary outcome in the current study. For future clinical trials, it would be very helpful if outcomes are developed that have high clinical relevance and are proved to be related to improvements observed in preclinical studies leading up to these clinical trials.

Finally, translation could be affected by the finding that cognitive and behavioural deficits in individuals with NF1 have been linked to structural brain alterations that are not present in the *Nf1* mouse model. These brain alterations include T2‐weighted hyperintensities, volumetric changes, and microstructural and connectivity alterations.^56^ The interdependency of these aetiological factors in relation to the cognitive challenges for individuals with NF1 needs further investigation, which could reveal whether certain radiological biomarkers are predictive of treatment response and should be used as an exclusion criterion in NF1 trials.

In conclusion, our findings do not provide evidence for a positive effect of lamotrigine on cognitive function in adolescents with NF1. The strengths of the current study were the high compliance to treatment, low drop‐out rate, and clinically relevant outcomes. The limitations of this study were the small sample size, an imbalance in diagnoses of autism spectrum disorder between groups, and potentially concomitant methylphenidate use. Nevertheless, the small effect sizes – particularly on the primary outcome (performance IQ) – strongly indicate that the current trial would still yield a negative result regardless of these limitations. For preclinical studies that investigate the age‐dependent effect of lamotrigine, assessment on neurocognitive outcomes in a younger study population with NF1 could be considered. However, we do not recommend further investigating lamotrigine as a (mono)treatment for improving cognitive function in adolescents with NF1.

## FUNDING INFORMATION

This study was financially supported by Let's Beat NF, The Netherlands Organization for Health Research and Development (ZonMw) (grant number 40–41900–98‐224) and the De Stichting Sophia Kinderziekenhuis Fonds (grant number B14‐02). Furthermore, Teva pharmaceuticals sponsored this study by providing the study drug.

## CONFLICT OF INTEREST STATEMENT

MCW has acted in the advisory board of Jazz Pharmaceuticals (compensation paid to the hospital) and has acted as the local PI for an industry driven study by Roche. Both activities were unrelated to this topic. RO has acted in the advisory board of Alexion (unpaid) and has received funding as a local investigator for research unrelated to this topic carried out by Alexion. EL receives occasional consulting fees from Alexion and Springworks Therapeutics.

## Supporting information


**Appendix S1:** Research protocol.

**Appendix S2:** Statistical analysis plan.

**Figure S1:** Trial profile.

**Table S1:** Alternative treatment effect models.

**Table S2:** Table of lamotrigine serum levels.

## Data Availability

The data that support the findings of this study are available from the corresponding author upon reasonable request.
