# Supplementary material for: Lamotrigine for cognitive deficits associated with neurofibromatosis type 1: A phase II randomized placebo‐controlled trial
Source: Dev Med Child Neurol. 2024 Sep 28;67(4):537–49. doi: 10.1111/dmcn.16094 (PMC11875526; doi:10.1111/dmcn.16094)
Supplement: Supplementary file 1 — Appendix S1: Research protocol. [file DMCN-67-537-s003.pdf]

RESEARCH PROTOCOL

The effect of lamotrigine on cognitive deficits associated with Neurofibromatosis type 1: a phase II randomized, placebo-controlled, multi-centre trial (NF1-EXCEL)

Version 9, December 7, 2018

NL 44912.078.13

Expertise centre ENCORE

Centre for Human Genetics

Department of Paediatrics

PROTOCOL TITLE:

'The effect of Lamotrigine on cognitive deficits associated with Neurofibromatosis type 1: a phase II randomized controlled multi-centre trial (NF1-EXCEL)'

**Protocol ID**	**NF1-EXCEL (Examining the Cognitive and Electrophysiological benefit of Lamotrigine in NF1)**
**Short title**	**Effect of Lamotrigine on cognition in NF1**
**Version**	**9**
**Date**	**2018-12-07**
**EUDRACT-number**	**2013-003405-26NL**
**Coordinating investigator**	**Dr. Marie-Claire de Wit (Neurology)**<br>**Contact: drs. M.J.Ottenhoff/ drs. J. Castricum**<br>**Expertise centre ENCORE, Room SP-1537**<br>**Dr. Molewaterplein 50**<br>**3015 GE Rotterdam**<br>**m.ottenhoff@erasmusmc.nl/**<br>**1.1.1 j.castricum@erasmusmc.nl**<br>**tel: 010-703 4969**
**Erasmus MC – Site Leader**	**Dr. Marie-Claire de Wit**
**UZ Leuven – Site Leader**	**Prof.dr. Eric Legius**<br>**eric.legius@uzleuven.be**
**HSJD Barcelona –Site Leader**	**Dr. Hector Salvador Hernandez**<br>**hsalvador@sjdhospitalbarcelona.org**
**Project leaders**	**Prof.dr. Ype Elgersma (Neuroscience)**<br>**Prof.dr. Henriëtte Moll (Paediatrics)**
**Sponsor:**	**Erasmus MC – Rotterdam**
**Independent physician(s)**	**Drs. G.C.B. de Heus - Bindels (Paediatrics)**
**Laboratory sites**	**Erasmus MC – Pharmacy**
	**Erasmus MC – Clinical chemistry**

Erasmus MC – Department of Psychiatry

PROTOCOL SIGNATURE SHEET (FOR SITE: ERASMUS MC)

Name	Signature	Date
**Dr. M.C.Y. de Wit**<br><br>***Department of Neurology, site leader***		
**Dr. J.H.M. Tulen**<br><br>***Department of Psychiatry, investigator***		
**Prof.**<br><br>**dr. H.A. Moll**<br><br>***Co-principal investigator***		
**Prof. dr. Y. Elgersma**<br><br>***Co-principal investigator***		
**Prof. dr. P.A.E. Sillevis Smitt**<br><br>***Head of Department of Neurology***		
**Prof. dr. E.H.H.M. Rings**<br><br>***Head of Department of Paediatrics***		
**Prof. dr. C.I. de Zeeuw**<br><br>***Head of Department of Neuroscience***		
**Prof. dr. W.J.G. Hoogendijk**<br><br>***Head of Department of Psychiatry***		

TABLE OF CONTENTS

1. INTRODUCTION AND RATIONALE 10
   1.1 Cognitive deficits associated with NF1 10
   1.2 Previous studies into drug treatment of cognitive deficits in NF1 10
   1.3 Detailed mechanistic insight 11
   1.4 Lamotrigine as a potential disease-modifying drug 12
   1.5 Rationale for selecting outcome measurements 13
   1.6 Safety and burden of transcranial magnetic stimulation 15
2. OBJECTIVES 16
3. STUDY DESIGN 17
   3.1 Study design 17
   3.2 Setting 17
   3.3 Schedule (next page) 18
   3.4 Intervention 20
   3.5 Measurements 20
4. STUDY POPULATION 21
   4.1 Population (base) 21
   4.2 Inclusion criteria 21
   4.3 Exclusion criteria 21
   4.4 Medical examination 22
   4.5 Sample size calculation 22
5. TREATMENT OF SUBJECTS 23
   5.1 Investigational product/treatment 23
   5.2 Use of co-intervention (if applicable) 23
6. INVESTIGATIONAL MEDICINAL PRODUCT 25
   6.1 Name and description of investigational medicinal product(s) 25
   6.2 Summary of findings from clinical studies 25
   6.3 Summary of known and potential risks and benefits 25
   6.4 Description and justification of route of administration and dosage 25
   6.5 Dosages, dosage modifications and method of administration 27
   6.6 Preparation and labeling of Investigational Medicinal Product 27
   6.7 Drug accountability 27
7. METHODS 28
   7.1 Study parameters/endpoints 28
      7.1.1 Main study parameter/endpoint 28
      7.1.2 Secondary study parameters/endpoints 28
      7.1.3 Process measurements 28
   7.2 Randomisation, blinding and treatment allocation 29
   7.3 Study procedures 29
   7.4 Withdrawal of individual subjects 34
   7.5 Follow-up of subjects withdrawn from treatment 34
   7.6 Premature termination of the study 34

8. SAFETY REPORTING 36
8.1 Section 10 WMO event 36
8.2 Adverse and serious adverse events....................36
8.2.1 Suspected unexpected serious adverse reactions (SUSAR) 37
8.2.2 Annual safety report 38
8.3 Follow-up of adverse events....................38
8.4 Data Safety Monitoring Board (DSMB) 38
9. STATISTICAL ANALYSIS 39
ETHICAL CONSIDERATIONS 40
9.1 Regulation statement 40
9.2 Recruitment and consent....................40
9.3 Benefits and risks assessment, group relatedness 40
9.4 Compensation for injury 41
9.5 Incentives....................42
10. ADMINISTRATIVE ASPECTS AND PUBLICATION 43
10.1 Handling and storage of data and documents 43
10.2 Amendments....................43
10.3 Annual progress report....................43
10.4 End of study report....................44
10.5 Public disclosure and publication policy....................44
11. REFERENCES 45

LIST OF ABBREVIATIONS AND RELEVANT DEFINITIONS

ABR	ABR form, General Assessment and Registration form, is the application form that is required for submission to the accredited Ethics Committee (In Dutch, ABR = Algemene Beoordeling en Registratie)
AE	Adverse Event
AR	Adverse Reaction
CA	Competent Authority
CCMO	Central Committee on Research Involving Human Subjects; in Dutch: Centrale Commissie Mensgebonden Onderzoek
CV	Curriculum Vitae
DSMB	Data Safety Monitoring Board
EU	European Union
EudraCT	European drug regulatory affairs Clinical Trials
GCP	Good Clinical Practice
IB	Investigator’s Brochure
IC	Informed Consent
IMP	Investigational Medicinal Product
IMPD	Investigational Medicinal Product Dossier
METC	Medical research ethics committee (MREC); in Dutch: medisch ethische toetsing commissie (METC)
NF1	Neurofibromatosis type 1
(S)AE	(Serious) Adverse Event
SPC	Summary of Product Characteristics (in Dutch: officiële productinfomatie IB1-tekst)
Sponsor	The sponsor is the party that commissions the organisation or performance of the research, for example a pharmaceutical company, academic hospital, scientific organisation or investigator. A party that provides funding for a study but does not commission it is not regarded as the sponsor, but referred to as a subsidising party.
SUSAR	Suspected Unexpected Serious Adverse Reaction
TMS	Transcranial Magnetic Stimulation
Wbp	Personal Data Protection Act (in Dutch: Wet Bescherming Persoonsgevens)
WMO	Medical Research Involving Human Subjects Act (in Dutch: Wet Medisch-wetenschappelijk Onderzoek met Mensen

SUMMARY

Rationale: Neurofibromatosis type I (NF1; incidence 1:3000) is one of the most common monogenetic causes of cognitive disability. It is an autosomal dominant disorder, caused by mutations in the NF1 gene, and characterized by a wide variability of cutaneous manifestations, neurofibromas, and cognitive, social, motor and emotional problems. Despite the frequency of the disorder and the impact on daily life, there is currently no evidence-based treatment targeting the cognitive problems in NF1. The ENCORE-laboratory at Erasmus MC has recently shown that the cognitive deficits in *Nf1* mice are caused by attenuated function of HCN-channels (hyperpolarization-activated cyclic nucleotide-gated channels) in interneurons. In mice, lamotrigine (LTG), an HCN-sodium channel agonist, rescues the neuronal plasticity and learning deficits. lamotrigine is approved to treat epilepsy and bipolar disorder, and is frequently used in children with and without NF1 to treat epilepsy. We hypothesize that lamotrigine will improve neuronal plasticity in adolescents with NF1 and improve their cognitive functioning.

Objective: The objective of this proposal is to determine the effect of lamotrigine on cognitive functioning and neurophysiology in adolescents with NF1.

Study design: Phase II randomized double-blind placebo-controlled parallel group multi-centre trial

Study population: 60 patients with genetically confirmed NF1 aged 12-17.5 years.

Intervention: Lamotrigine or placebo tablets: period of 8 weeks dose increase to the target dose of 2 x 100 mg/d, followed by 18 weeks of target dose treatment.

Main study parameters/endpoints:

Primary outcome measure: Difference in standard scores on total performal scale of the Wechsler intelligence scales after 6 months of treatment, adjusted for baseline performance.

Secondary outcome measures: neuropsychological tests of visual-spatial learning efficacy, motor functioning, visual perception and attention. Questionnaires by parents on attention and executive functions. Neurophysiological correlates of synaptic and cortical plasticity as underlying mechanisms of cognitive function: Intra-cortical inhibition of the M1 motor cortex; and plasticity of motor cortex.

Nature and extent of the burden and risks associated with participation, benefit and group relatedness:

Burden: Participants are required to take lamotrigine or placebo tablets twice daily for 28 weeks. They have to keep a patient diary. Neuropsychological tests are assessed at baseline and after 6 months and non-invasive neurophysiology measures at baseline and 10 weeks. The participants will visit the outpatient clinic four times: at T= -1 week, T=0 weeks, T=10 weeks end T=26 weeks. A home visits is made at T=18 weeks to monitor compliance and

adverse events. Between the visits, parents and participants will be contacted by telephone at time points 4, 8, 14, and 22 weeks. After T=26, there is a build-off phase of 2 weeks, after which there is an extra telephone contact at T= 28 weeks. At T=52 weeks, participants receive a questionnaire to monitor off-phase attention problems. Finger prick blood will be obtained at T=-1 week to assess renal function, liver enzymes and complete blood count, at T=10 to monitor lamotrigine blood levels, liver enzymes and complete blood count and at both T=18 and T=26 to monitor lamotrigine blood levels. Total time investment by the participants for visits and testing will be +/- 11 hours.

Risks: Side-effects that are associated with lamotrigine are known, manageable and subside upon dose reduction or withdrawal of the study medication (see SPC). Special attention is directed at the occurrence of skin rash, which can be severe if medication is continued and which will therefore result in immediate withdrawal of study medication. There are no specific risks associated with outcome assessments. TMS, performed by trained personnel, is a non-invasive and safe method of measuring cortical inhibition and plasticity.

Benefit: If lamotrigine has a positive effect on the cognition of adolescents with NF1, participants will have direct benefit of participating in this trial. In addition, we would establish high grade evidence for treatment of cognitive deficits in a vulnerable paediatric population.

Group relatedness: There are several reasons to perform this study in children/adolescents instead of adults. These reasons are similar to trials previously performed in this population (MEC-2005-281 and MEC-2009-086).

We expect the children's/adolescent's brain to have the highest ability to change (most 'plasticity'). Issues in daily life related to cognitive and behavioural deficits are most prominent in children/adolescents.

- The potential benefit of this study will directly apply to the study population.
- This intervention is aimed at a NF1 specific neuronal dysfunction, so that testing of healthy volunteers cannot produce data that NF1 patient will benefit from.
- NF1-children/adolescents have a very characteristic profile of problems with school performance, behaviour and cognition. In contrast, at adult age, these problems have accumulated to a mild, broad range of cognitive deficits that are harder to quantify (probably because of adapting alternative problem-solving strategies and avoidance of learning environments such as school).
- From a practical point of view, we can only use some of the outcome measures (e.g. some of the attention tasks, parent-rated questionnaires) in children/adolescents.
- Again from a practical point of view, there are no specific outpatient clinics for adult NF1 patients and the patients that are available often suffer from complex somatic complications of NF1, reducing generalizability of the results of this trial. In contrast, the outpatient clinics of the Sophia's Children's Hospital, UZ Leuven and KBO-Kinderzentrum

München / Technische Universität München, Munich are representative of children with NF1 because the clinics are easily accessible and children remain in yearly follow-up after diagnosis.

- Finally, the lower limit of 12 years is chosen, as dosages under this age limit are calculated according to body weight (kilograms) and will result in a large number of different dosages. In this proof-of principle study it is not feasible to produce placebos for every possible dosage.

2. INTRODUCTION AND RATIONALE

2.1 Cognitive deficits associated with NF1

Neurofibromatosis type 1 is among the most common (1:3000) single-gene disorders causing cognitive disabilities. Children with NF1 have a lower mean IQ, specific deficits in visual-spatial skills, nonverbal long term memory, executive functions, attention and motor performance [1]. NF1 also has a large impact on school performance [1]. In total, 75% of patients have learning disabilities (school performance more than 1 standard deviation below average). 37% of the Dutch children with NF1 are in schools for the learning disabled (special education) and another 48% need remedial teaching. Due to the lower education level, the socio-economic status of families with a NF1-parent is below average [2, 3]. Behavioural problems also occur more often. Children with NF1 display more internalizing behavioural problems, like social withdrawal, anxiety and depressive symptoms [2, 3]. 40% of NF1-children meet diagnostic criteria for ADHD, which is at least three times more than the general population [1, 4]. As expected, NF1 severely affects quality of life in motor, cognitive, social and emotional domains, as reported by NF1-children and their parents [3].

2.2 Previous studies into drug treatment of cognitive deficits in NF1

This randomized trial is the third clinical trial into the effect of a drug on cognitive deficits in children/adolescents with Neurofibromatosis type 1 coordinated by Expertise centre ENCORE at Erasmus MC. The first two were carried out with simvastatin, a cholesterol lowering drug, aimed at targeting activated RAS-proteins. Testing statins in the clinic for treatment of cognitive deficits in children with NF1 was very worthwhile for the following reasons. First, statins influence the activity of the RAS-protein, as it is hypothesized that they lower farnesyl anchors. The RAS-protein needs farnesyl, as it can only be active when it is attached to the cell membrane by such an anchor. Second, administrating statins to *Nf*1 mice, rescued their learning and memory deficits. Finally, statins are known for their favourable side effect profile.

The first trail conducted was a 12-week study, which showed improvements on the Object Assembly task (WISC-III-NL), a secondary outcome, but did not show improvement on primary outcomes of visual spatial memory, attention, motor learning or MRI-abnormalities (MEC-2005-281). In the trial report, we concluded that a trial with longer treatment duration was warranted [5]. The second trial, NF1-SIMCODA (MEC-2009-086), was designed to establish the therapeutic effects of simvastatin after a 12-month treatment period [6]. This study showed that simvastatin had no effect on primary outcomes of cognition, attention, internalizing behavioural problems or any of the secondary outcome measures. Combining data from both trials, we conclude that simvastatin is not effective in improving cognitive and behavioural deficits in NF1.

The reason for these negative results and not succeeding in translating the preclinical findings to the clinic could have several reasons. Possibly, simvastatin cannot pass the blood brain barrier or influence the activity of RAS as well in humans compared to mice. Other candidate drugs, preferentially drugs that are more specifically targeted towards the underlying mechanism, should be tested.

What we can conclude from the successful completion of two of such studies is that cognitive deficits are indeed a reason for parents and patients to participate in long-term medication trials. In addition, our setting is optimized at performing such studies.

2.3 Detailed mechanistic insight

Using *Nf1* mutant mice, it has previously been shown that the cognitive deficits in *Nf1* mice are caused by increased inhibition from inhibitory interneurons [7-10]. Clinical evidence further supports this [9]. We have now identified the mechanism for this increased inhibition. We identified HCN1 as an NF1-interacting protein, and show that a selective attenuation of hyperpolarization-activated cyclic nucleotide-gated (HCN) current in parvalbumin-expressing (PV) interneurons is the cause for increased inhibition in *Nf1* mutants. Specifically, we provided 4 independent lines of evidence. First, HCN currents are selectively reduced in interneurons of *Nf1* mice, which increases their excitability. Second, blocking HCN channels mimics the enhanced inhibition as observed in loss-of-function *Nf1* mice. Third, the electrophysiological deficits of *Nf1* mutant mice are rescued by the clinically-approved medication, lamotrigine, a potent HCN agonist. Finally, and highly encouraging of the human translational potential of our findings, we found that lamotrigine also fully rescues the learning deficits of two different NF1 mouse models, and the motor learning deficits in the classical *Nf1* mouse model. These results are now under review for publication (Nature Neuroscience). Since the data are not yet published, we have attached some of the key figures of the manuscript to the protocol (figure 1).

2.4 Lamotrigine as a potential disease-modifying drug

Lamotrigine is an attractive candidate for use as a disease modifying drug in NF1-patients for the following reasons.

First, the preclinical experiments explained in the paragraph above show that the biomedical rationale is strong. These experiments elucidate that lamotrigine is a more specific and potent treatment cognitive problems in NF1 compared to statins. Lamotrigine specifically targets the inhibitory interneurons, which are proven to be selectively impaired in NF1 by targeting HCN1. While statins target both excitatory and inhibitory neurons.

Second, lamotrigine has already proven its effectiveness in treating diseases of the central nervous system. It is FDA/EMEA approved for epilepsy and bipolar disorder and is considered effective in these disorders because of being a HCN-agonist [11], the same property of lamotrigine we propose to be effective in treating cognitive problems in NF1. Additionally, in world-wide practice, lamotrigine is used in patients with NF1 that have comorbid epilepsy. Also, neurologists report a better effect on behaviour in children that receive lamotrigine compared with other anti-epileptic drugs [12].

Finally, lamotrigine has a known safety profile (see chapter 6 of this protocol and section D of the study documentation). The lead clinician in this study (dr. M.C.Y. de Wit, neurologist) has extensive experience in the use of lamotrigine in children with epilepsy and in children with NF1 and epilepsy. Under appropriate clinical supervision, use of lamotrigine is considered safe and side-effects that occur can be managed.

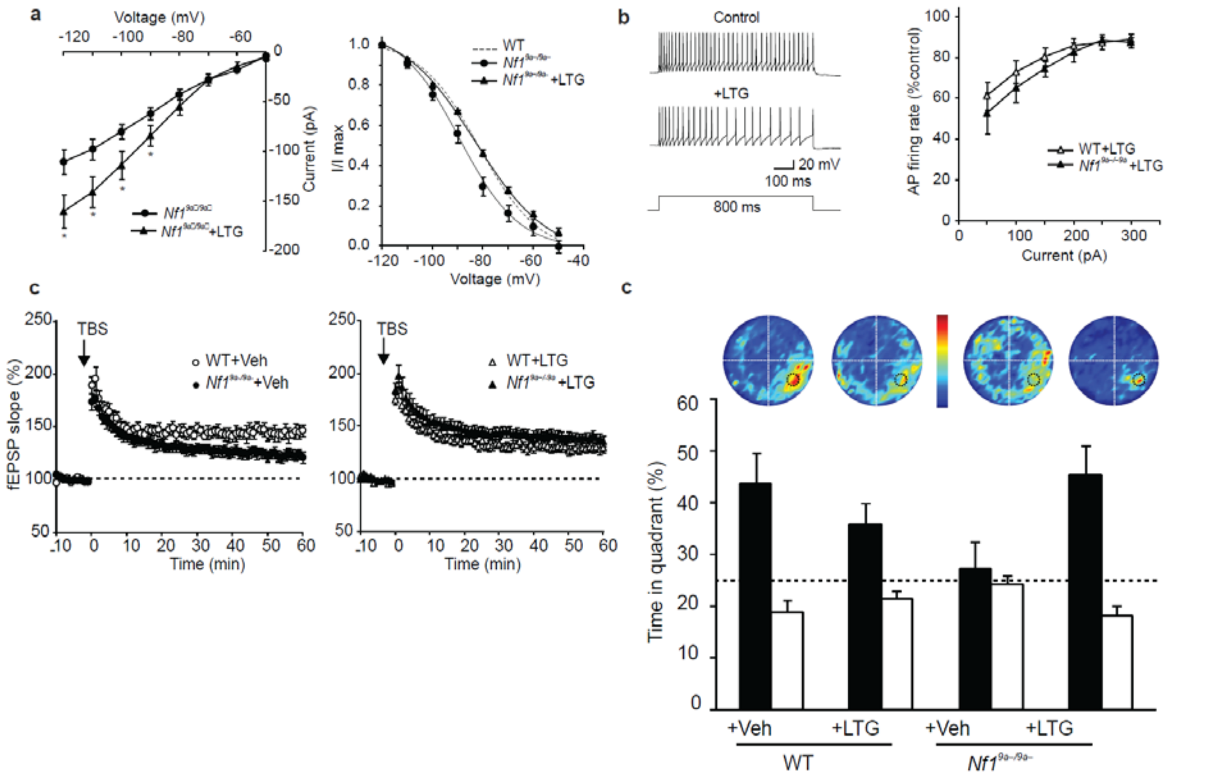

Figure 1: Learning deficit of Nf1-mice is rescued by lamotrigine. A) Current through HCN-channels in *Nf1*-mice reaches levels of the control mice, as measured by whole cell patch clamp in inhibitory interneurons; B) Lamotrigine rescues the higher number of action potentials in inhibitory interneurons in *Nf1*-mice. C) Black bars represent the percentage of time in the target quadrant of the Morris Watermaze, a spatial learning task. Higher bars mean better learning. Lamotrigine rescues the learning deficit in *Nf1*-mice (From Omrani et al., under review, full manuscript available if requested).

2.5 Rationale for selecting outcome measurements

Since improving cognition in NF1 has not been achieved before, it is unclear what outcome measures would show an effect most clearly. We chose the outcome measures of this trial to cover three areas of the broad area of cognitive deficits in NF1. They are based on our extensive experience in clinical trials in this population.

The primary outcome measure of performal intelligence is chosen for its clinical relevance: intelligence informs us on the clinical benefit of lamotrigine treatment, since IQ is highly correlated with school performance, daily life functioning, and later socio-economic status. In addition, the intelligence scales that we use (the WISC-III and WAIS-III, Dutch or German version) have excellent psychometric properties. Specifically, their test-retest reliability makes the test useful for clinical trials. As stated above, the IQ-distribution of individuals with NF1 is shifted to the left, meaning a significantly lower IQ in individuals with NF1 compared with sibling or healthy population controls.

The first set of secondary outcome measures are five short psychological tests and two questionnaires (for parents) that measure the specific deficits that are observed in NF1-patients, and that share analogy with tests used in animal models of NF1. These are tests for visual-spatial learning efficacy, motor functioning, visual perception and attention (see methods). These tests aim to evaluate the psychological domains through which lamotrigine might improve overall cognitive functioning. Neuropsychological testing is done before treatment and after 26 weeks of treatment. This time frame is chosen since repeating neuropsychological testing in a shorter time frame reduces the reliability of most tests, for example by increasing training effects.
A second set of secondary outcome measures consist of non-invasive measurements of cortical inhibition and neuronal plasticity through TMS. These can be used as a biomarker for the reduction in inhibition and increase in neuronal plasticity, potentially mediated by increased N-methyl-d-aspartate (NMDA) receptor currents [13] and decreased intracortical inhibition. The hypothesis is that the target dose (2 x 100 mg) of lamotrigine will lead to immediate effects on HCN-channels, intracortical inhibition and synaptic plasticity. This is supported by data from the animal model, see above, and data from research in healthy human volunteers, that showed changes in TMS-measures after single high dose administration [14]. Therefore, TMS is measured before administration of lamotrigine (T=0) and at time point of maximal dosage (T=10). For ethical reasons of time investment, TMS is not measured at the endpoint of lamotrigine administration (T=26 weeks). It is very important to include these measures, since this adds information on the biological activity of the drug and provides a mechanistic insight in the physiologic pathways through which lamotrigine works in NF1-patients. Without it, results from the psychological tests are more difficult to interpret. It will also allow us to search for markers to predict and monitor individual response in later trials or in clinical treatment. At timepoint T=10, the TMS-endpoint measurement is combined with a physical exam and an ADHD-questionnaire, to reduce the number of hospital visits.

In total, we have defined one primary outcome measure and nine secondary outcome measures. We have chosen to restrict the number of outcome measures as much as possible, both to minimize the burden for the participants, as well as to prevent inflation of the chance of false-positive findings. The secondary outcome measures serve to cover all neuropsychological subdomains potentially relevant to explain the effect of lamotrigine on the primary outcome measure (general performal intelligence) and to inform future clinical use and/or studies.

2.6 Safety and burden of transcranial magnetic stimulation

Transcranial magnetic stimulation (TMS) is a non-invasive way to evaluate the function of the human cortex. It is considered to be safe for children, adolescents and adults [15, 16]. TMS has two main applications. The first is as a diagnostic tool to evaluate excitability, inhibition and plasticity of the motor cortex. This is the application we use in this protocol. Our protocols make use of single-pulse and paired-pulse paradigms that do not carry an elevated risk of adverse events above the level of normal daily life.

The second application of TMS is treatment of depressive disorders. For treatment purpose, the cortex is stimulated with high frequency and suprathreshold (i.e. intensities above active motor threshold) stimulation intensity over prolonged periods of time: conventional repetitive TMS (rTMS). These intensive treatment protocols carry a slightly elevated risk of side effects, including headache and elevated chance of epilepsy. We would like to stress that we will not use treatment, using suprathreshold rTMS, protocols in this study.

We regard the use of single- or paired pulse TMS for research purposes a minimal-risk procedure, comparable to magnetic resonance imaging (MRI) and venipuncture. Exclusion criteria are defined according to the safety guidelines of [15] thereby further minimizing the risk of adverse events. The burden consists of sitting calm in a comfortable chair during stimulation. The stimulation with the TMS-coil is painless and feels like tapping on the head with a finger. Electromyographic recordings are performed with non-invasive surface electrodes (no needles) on the left thumb muscle. For induction of neuronal plasticity, we aim to use the well-established and safe “paired associative stimulation” (PAS) protocol combining a central magnetic stimulus with a weak (slightly above perception threshold) electrical stimulus at the wrist. This stimulation refers to model of associative plasticitiy in human M1. It is our experience that children and adolescents with NF1 (the age group in this protocol) are able to undergo measurements with a similar setup, for instance prism adaptation measurements or MRI [5]. The collaborating centre at Technische Universität München, led by prof. Volker Mall, has recently published several studies including adults with NF1 [17], a study including adolescent patients with Noonan syndrome (a patient group comparable to NF1 patients in terms of cognitive deficits) [18] and children/adolescents with autism spectrum disorders [19]. Considerations, relevant to ethical approval, on the use of single- and paired pulse TMS in children have been published [20]. The authors of that paper conclude that TMS, as used in this protocol, carries no greater risk than MRI, a procedure frequently applied to children in research projects, both therapeutic and non-therapeutic.

3. OBJECTIVES

The objective of this proposal is to find proof-of-principle for an effect of lamotrigine on cognitive functioning in adolescents with Neurofibromatosis type 1.

Secondary objectives are the evaluation of the safety and the effect of lamotrigine on subdomains of cognitive function, intra-cortical inhibition and LTP-like plasticity.

4. STUDY DESIGN

4.1 Study design

Randomised, placebo-controlled, double-blind, parallel-group clinical trial.

4.2 Setting

This study will be performed within Erasmus MC – ENCORE Expertise centre for neurodevelopmental disorders and four other European clinical research centres, i.e. University Hospital Leuven, Belgium; KBO-Kinderzentrum/Technische Universität München, Germany; University of Manchester (Institute of Brain, behaviour and Mental Health), United Kingdom; and University of Padova, Italy.

ENCORE is a collaboration between clinical and fundamental Erasmus MC – departments, aiming at a better understanding of cognitive genetic disorders and better care for individuals with these disorders. Departments that are primarily involved in this protocol are the departments of Neurology, Neuroscience and Paediatrics (see table of specialists involved). Participants will be recruited from the ENCORE - NF1 Expertise centre (see patient section), which has a longstanding history of research and care in NF1.

One of the principal investigators, Prof. Elgersma of the department of Neuroscience, has identified the proposed treatment in mouse models, and is also the scientific director of ENCORE. At this expertise centre, several clinical trials have been successfully completed, are currently in progress or in early stages of planning.

The main principal investigator (Dr. De Wit) is involved in several clinical trials within ENCORE and has extensive clinical experience with prescribing Lamotrigine as a paediatric neurologist. In addition, she is familiar with NF1-patients in the clinic.

Our collaborators at KBO-Kinderzentrum/Technische Universität München have extensive experience with transcranial magnetic stimulation in individuals with NF1, Noonan syndrome, autism and many other disorders. Our collaborators in Leuven have successfully joined us in the previous clinical trial in children with NF1 and are an internationally famous centre on research in NF1. Erasmus MC - staff involved in this protocol:

Name	Appointment	Department	Role in this proposal
Dr. M.C.Y. de Wit	Neurologist, Child-neurologist	Neurology	Principle investigator; Medical expert on Lamotrigine and NF1-patient care
Prof.dr. Elgersma	Professor	Neuroscience	Supervision of study
Prof.dr. Moll	Professor	Paediatrics	Supervision of study

Dr. R. Oostenbrink	Paediatrician	Paediatrics	Coordinator of national NF1 referral centre in NL
Drs. A.B. Rietman	Neuropsychologist	Neurology	Neuropsychological testing
Drs. M.H.T. van der Vaart	PhD-student	ENCORE (Neuroscience/Pediatrics/ Neurology)	Study design, study preparation
Drs. M.J. Ottenhoff	PhD-student/ research physician	Pediatrics/Neuroscience/ Neurology	Daily execution of the trial, study preparation, study design
Dr. J.H.M. Tulen	Associate Professor of Psychophysiology	Psychiatry	TMS-expert at Erasmus MC
Dhr. B. Manai	Research Nurse	Pediatrics	Compliance and safety; monitoring

The steering committee of this study will consist of the following persons:

Prof.dr. Y. Elgersma

Prof.dr. E. Legius

Prof.dr. V. Mall

Prof.dr. H.A. Moll

Dr. M.C.Y. de Wit

Dr. J.H.M. Tulen

Drs. T. van der Vaart

Drs. M.J. Ottenhoff

4.3 Schedule (next page)

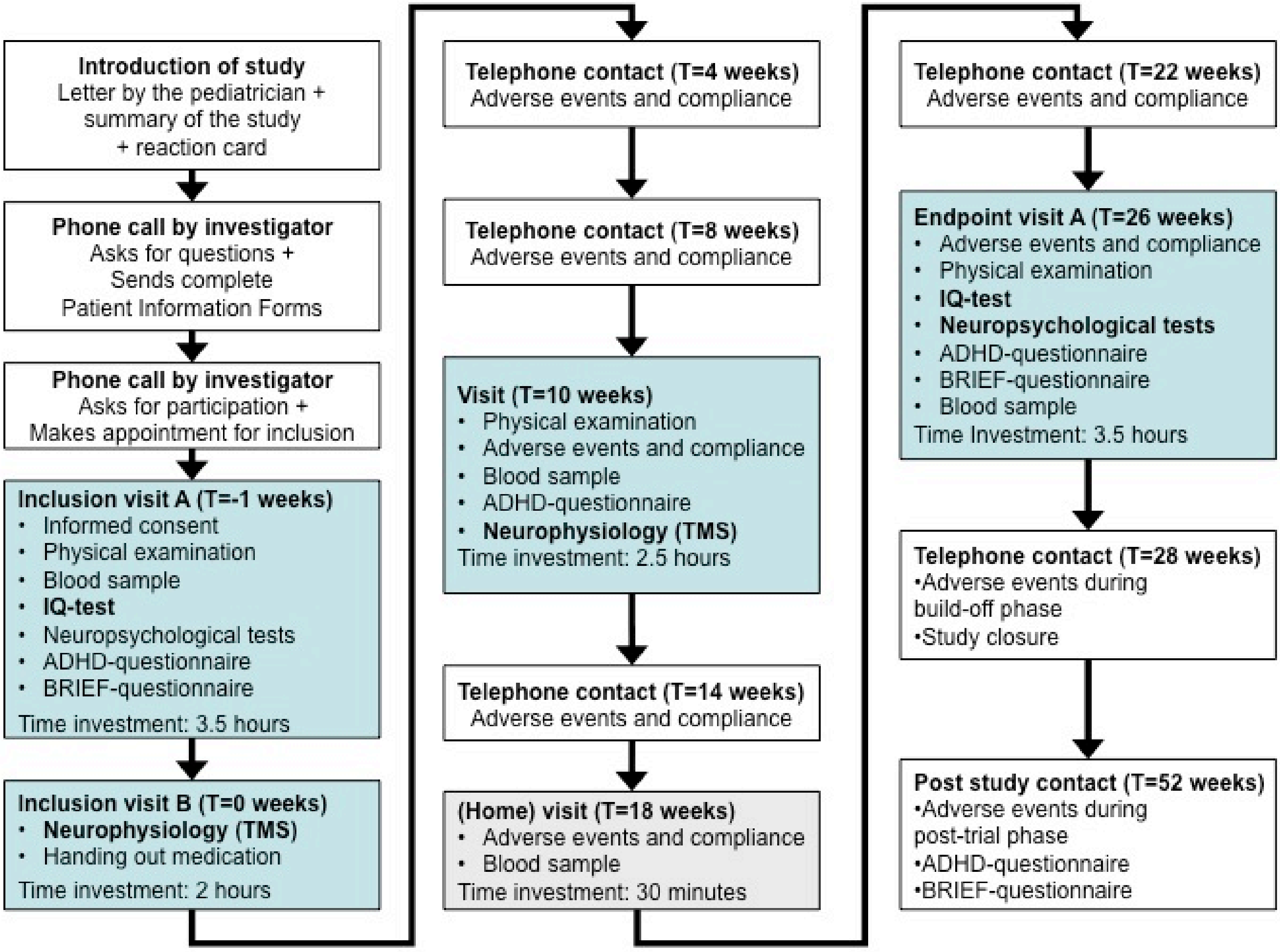
Introduction of study
Letter by the pediatrician + summary of the study + reaction card
Phone call by investigator
Asks for questions + Sends complete Patient Information Forms
Phone call by investigator
Asks for participation + Makes appointment for inclusion
Inclusion visit A (T=-1 weeks)
• Informed consent
• Physical examination
• Blood sample
• IQ-test
• Neuropsychological tests
• ADHD-questionnaire
• BRIEF-questionnaire
Time investment: 3.5 hours
Inclusion visit B (T=0 weeks)
• Neurophysiology (TMS)
• Handing out medication
Time investment: 2 hours
Telephone contact (T=4 weeks)
Adverse events and compliance
Telephone contact (T=8 weeks)
Adverse events and compliance
Visit (T=10 weeks)
• Physical examination
• Adverse events and compliance
• Blood sample
• ADHD-questionnaire
• Neurophysiology (TMS)
Time investment: 2.5 hours
Telephone contact (T=14 weeks)
Adverse events and compliance
(Home) visit (T=18 weeks)
• Adverse events and compliance
• Blood sample
Time investment: 30 minutes
Telephone contact (T=22 weeks)
Adverse events and compliance
Endpoint visit A (T=26 weeks)
• Adverse events and compliance
• Physical examination
• IQ-test
• Neuropsychological tests
• ADHD-questionnaire
• BRIEF-questionnaire
• Blood sample
Time Investment: 3.5 hours
Telephone contact (T=28 weeks)
•Adverse events during build-off phase
•Study closure
Post study contact (T=52 weeks)
•Adverse events during post-trial phase
•ADHD-questionnaire
•BRIEF-questionnaire

4.4 Intervention

Participants will be randomized to lamotrigine or placebo, using computer generated permuted block randomization lists generated by the department of biostatistics. The hospital pharmacist will perform randomization. For details on the drug dosing, see chapter 6.

4.5 Measurements

Neuropsychological testing will be performed using: 1) the Wechsler Intelligence Scales for Children (WISC) – III NL or Wechsler Adult Intelligence Scales (WAIS)-IV-NL; 2) Cambridge Neuropsychological test Automated battery (CANTAB) – Paired Associate Learning task; 3) Motor free Visual Perception Test (MVPT); 4) Amsterdam Neuropsychological Tasks (ANT) – Sustained Attention DOTS task; 5) Grooved Pegboard test; 6) Beery Visual Motor Integration (Beery-VMI) – motor coordination test.

Questionnaires will be taken using: a) the parent-reported ADHD-questionnaire AVL, for assessing attention problems; b) the parent-reported BRIEF-questionnaires for assessing executive function.

Intracortical inhibition will be measured using transcranial magnetic stimulation – paired pulse paradigm.

LTP-like plasticity will be measured using transcranial magnetic stimulation paired with peripheral nerve stimulation (paired associative stimulation, PAS).

Lamotrigine blood levels will be obtained tree times per participant: a through level and levels at 3 hours (at expected T_{max}) and 6 hours post dose. Simultaneously body weight, height, time of last tablet intake and dosage, time of blood sampling and time after last consumption will be collected. Results of the lamotrigine blood levels will be disclosed after study completion of all participants.

Other measurements include blood safety measures and adverse events registration.

5. STUDY POPULATION

5.1 Population (base)

Participants are adolescents (12 years and older) with Neurofibromatosis type 1, recruited from the ENCORE – NF1 expertise centre at Erasmus MC. It is the national referral centre for NF1 patients with 27 years of experience and follow-up of paediatric patients with NF1. In addition, there is a close relationship with the patient organization (NFVN), and we will recruit subjects through NFVN as well.

5.2 Inclusion criteria

- NF1 patients with a genetically confirmed diagnosis.

  Only applicable for the Barcelona site: genetically or clinically confirmed diagnosis following strictly the NIH criteria. Participants with pigmentary only features (more than six café-au-lait spots or freckling in the axillary or inguinal regions), will have a genetically confirmed diagnosis according to the AACR guidelines [25]. In case the only non-pigmentary features are Lisch nodules, this will be confirmed by the ophthalmologist. A clinical diagnosis is based on presence of two of the following: 1) Six or more café-au-lait macules over 5mm in diameter in pre-pubertal individuals and over 15mm in greatest diameter in post-pubertal individuals. 2) Two or more neurofibromas of any type or one plexiform neurofibroma. 3) Freckling in the axillary or inguinal regions. 4) Two or more Lisch nodules (iris hamartomas). 5) Optic glioma. 6) A distinctive osseous lesion such as sphenoid dysplasia or thinning of long bone cortex, with or without pseudarthrosis. 7) First-degree relative (parent, sibling, or offspring) with NF-1 by the above criteria.
- Age 12-17.5 years at inclusion
- Oral and written informed consent by parents and assent from participants

5.3 Exclusion criteria

- Segmental NF1
- Severe hearing problems or deafness
- Severe visual problems or blindness
- Use of the following medication: fenytoïn, carbamazepine, fenobarbital, primidon, rifampicine, atazanavir/ritonavir, lopinavir/ritonavir, oxcarbazepine, topiramate, oral contraceptive pill (oestrogen and progestagen) and valproic acid during the last 3 months.
- Previous use of lamotrigine
- Previous allergic reactions to anti-epileptic drugs

- Epilepsy or epilepsy in the past
- Suicidal thoughts or behaviour
- Renal insufficiency
- Liver insufficiency
- Pregnancy
- Brain tumour or other brain pathology potentially influencing the outcome measures

5.4 Medical examination

During the screening process prior to inclusion, patients will undergo a medical examination. This includes a standard neurologic assessment. Kidney, liver function and complete blood count will be assessed by blood sample from finger prick and venipuncture at separate time points. Female NF1 patients will undergo a pregnancy test in urine. In addition, with the aid of a suicide behaviours questionnaire, patients will be assessed for possible suicidal thoughts and behaviour. This will be assessed, because neurotropic drugs, and anti-epileptic drugs in particular, have been shown to yield a higher occurrence of suicidal thoughts and behaviour [21, 22].

5.5 Sample size calculation

With an estimated test-retest reliability of 0.80 on the performal subscale of the Wechsler Scales for Intelligence and an alpha of 0.05, a sample size of 46 participants will have 80% power to detect an improvement of 0.5 SD (estimated around 7.5 performal IQ-points, which is considered clinically relevant). However, we decided to inflate the sample size to 60, to account for dropout-rate of 15% and a potential skewed number of participants between treatment arms, since randomization is stratified by centre, and it is uncertain whether randomization blocks will be perfectly filled. We will randomize 60 patients in a 1:1 ratio to Lamotrigine or placebo.

6. TREATMENT OF SUBJECTS

6.1 Investigational product/treatment

Lamotrigine tablets will be provided by TEVA Pharmaceuticals, Utrecht. *Only applicable for the Barcelona site:* Lamotrigine tablets of 100 mg will be provided by Mylan, Hertfordshire, UK. Details on these lamotrigine tablets can be found in the Summary of Product Characteristics (SPC) in Section D of the dossier. Placebo tablets will be identical in appearance and substance and produced by the pharmacy Haagsche Ziekenhuizen, The Hague. For the production of placebo tablets, the Investigational Medicinal Product Dossier (IMPD) is in preparation by the pharmacist. At time of submission of this protocol to the METC, this protocol was not finalized yet (see correspondence Erasmus MC hospital pharmacy in Section D of the dossier). *Only applicable for the Barcelona site:* Placebo tablets which will be comparable (but not identical) in appearance and substance are provided by the pharmacy Zentiva – Sanofi, Frankfurt, Germany.

6.2 Use of co-intervention (if applicable)

Lamotrigine is metabolised in the liver by glucuronidation. Co-application of lamotrigine with medication that result in an induction of glucoronidation leads to lowering of plasma levels by an uncertain amount. These substances include fenytoin, carbamazepine, fenobarbital, primidon, rifampicine, atazanavir/ritonavir and lopinavir/ritonavir. Therefore, subjects should not take these drugs prior and during the investigational treatment. In addition, the drug valproic acid significantly decreases glucoronidation of lamotrigine, and the combination will lead to increased plasma levels of lamotrigine. Therefore, use of valproic acid is an exclusion criterion. Other psychoactive drugs are also excluded for reasons of convergence on the pathway that is targeted, with the exception of methylphenidate (on the condition that no dosage increase or decrease occurred during a period of 3 months preceding inclusion). The use of oral contraceptive drugs can lower the plasma level of Lamotrigine with 50% by induction of glucuronyltransferase and show rebound effect during the stopping week. Thus, also the use of the oral contraceptive pill with a stopping week is an exclusion criterion. In emergencies and for any questions, the investigator will be available by phone for advice 24/7.

If it appears that a participant does have to take one of the above-mentioned drugs during the study, and no alternative treatment of first choice is available, he or she should be taken off lamotrigine in an appropriate manner and treatment delay by his/her participation in this study will be prevented by all means. If it appears that a patient eligible for this study is already taking one of the above-mentioned drugs, he/she will not be taken off this particular

drug to enable his/her inclusion in this study. If it appears that a participant is using methylphenidate and a dosage increase or decrease is needed during the study, he/she will remain in follow-up according to the intention-to-treat principle.

Although recommendations for use of lamotrigine allow for low doses of the drug during pregnancy, and the use of lamotrigine for epilepsy during pregnancy has proved to be associated with a low risk, we require female participants to use adequate contraception or abstain from intercourse (no oral contraceptive with stopping week, due to above interaction). In postmenarchal participants, a pregnancy test is required at inclusion visit A.

Effects of Lamotrigine on the ability to drive a car or to operate machines cannot be excluded, although investigations in healthy volunteers showed that lamotrigine has no different effect than placebo on fine motor coordination, eye movements or subjective sedative effects (see SPC). Therefore, participants will be asked for above signs, and will be counselled accordingly if necessary.

7. INVESTIGATIONAL MEDICINAL PRODUCT

7.1 Name and description of investigational medicinal product(s)

In this study, two investigational medicinal products are compared: lamotrigine and placebo. Lamotrigine is a marketed generic drug. For a description of lamotrigine tablets (TEVA/PCH), we refer to the Summary of Product Characteristics (SPC) in section D of the dossier. The tablets are provided by TEVA/PCH, not modified for this trial, other than the repackaging as described below. *Only applicable for the Barcelona site:* The 100 mg tablets are provided by Mylan, not modified for this trial, other than the repackaging as described below.

Placebo tablets are produced by pharmacy Haagsche Ziekenhuizen, The Hague, commissioned by the hospital pharmacy at Erasmus MC. There will be two types of placebo: 25 mg tablets and 100 mg tablets, comparable to the lamotrigine tablets. Erasmus MC pharmacy personnel (prof.dr. A. Vulto) will be responsible for batch release. *Only applicable for the Barcelona site:* Placebo tablets are provided by pharmacy Zentiva, Frankfurt, Germancy and will not be modified for this trial. There will be two types of placebo: 7 mm tablets and 10 mm tablets, comparable to the 25 and 100 mg lamotrigine tablets.

7.2 Summary of findings from clinical studies

A summary of findings from clinical studies with lamotrigine can be found in the SPC.

7.3 Summary of known and potential risks and benefits

For a complete overview of known and potential risks of lamotrigine: see SPC. Potential benefit in this study consists of improved cognitive functioning.

7.4 Description and justification of route of administration and dosage

A lamotrigine dose of 100 mg twice daily will be administrated orally.

Since this is the first study into the effect of lamotrigine on cognition in children with NF1, we aimed to choose a dose that is low enough to be safe and high enough to ensure the hypothesized effect on the brain can be measured. Presently, it is impossible to perform a dose finding trial for positive cognitive effects, since the outcome measures do not allow the minimal responsive dose estimation in small groups of participants over a short duration of time.

From a clinical perspective, maximal daily doses used for epilepsy treatment are up to 400-500 mg/d. Daily maintenance dose of 100-200 mg/d is advised for monotherapy of epilepsy. We (our collaborators in Munich) have observed effects on LTP-like plasticity in adult healthy volunteers using 300 mg [14]. A dose of 100 mg twice daily for adolescents, thus, seems appropriate.

Additionally, the chosen dosage corresponds very well to the human equivalent dose (HED). This HED could not be based on the half maximal effective concentration (EC50) in mice, as it is not possible to determine absolute values for the outcome measures used in the preclinical experiments, which identify a single animal as a responder. It is, however, possible to compare these measures for learning and memory between *Nf*1 mice and their wild type littermates. Several dosages were tested, of which 25 mg/kg turned out to be the most optimal for the following reasons. First, the administration of 25 mg/kg to *Nf*1 mice resulted in a full rescue compared to wild types. Second, a dosage of 10 mg/kg resulted in a partial effect. Finally, dosages exceeding 40mg/kg are associated with side effects in mice, and these were indeed observed at a dosage of 50mg/kg.

Therefore, the human equivalent dose (HED) was calculated, based on the 25 mg/kg dose in mice, according to international standards [23, 24]. We calculated HED's for the expected range and average body weight in our future study population (table below). This resulted in a HED-range of which 100 mg/kg is well in the middle.

We chose to administer this dosage twice a day because *Nf*1 mice show their rescued phenotype at T_{max}. Therefore, we aim to administer the HED as often as possible, without exceeding the currently known safe daily dosage for epilepsy (for the study population age group).

Oral administration of this dosage is justified, as both the bioavailability of lamotrigine by means of intraperitoneal injection in mice and oral administration in humans is very comparable, 100% and 98% respectively.

**HED calculation**						
Age (yr)	12		14,75		17,5	
SD	-2 SD		0 SD		+2 SD	
Sex	♂	♀	♂	♀	♂	♀
Body weight (kg) for SD, age and sex*	30		57	55	92	83
k_m**	29,89		37,42	36,95	44,25	42,68
**HED (mg)*** **	**75**		**114**		**145**	

** Based on normative data for the Dutch population.*

** $k_m = 9{,}09 \times W^{0{,}35}$

*** $HED\ (mg/kg) = animal\ dose\ (mg/kg) \times ((animal\ k_m)/(human\ k_m))$

In which animal k_m for mice is fixed at 3, and animal dose at 25 mg/kg.

7.5 Dosages, dosage modifications and method of administration

Film coated lamotrigine tablets will be administrated orally. Lamotrigine dose should be escalated according to the scheme provided by the manufacturer (see schedule next page). After the maintenance period and the outcome assessment, 100 mg lamotrigine a day will be given for two weeks to have a dose reduction period. It is unpractical to fabricate placebos for each possible dose during the dose escalation phase, therefore maximally three tablets of 25 mg have to be taken at one point in time, during a short duration of 2 weeks. During the main part of the trial, only one tablet has to be taken. The 25 mg tablets are small and we expect no difficulties for the subjects. Tablets can be swallowed as a whole or suspended in water. After the final end-visit, two weeks dose lowering will take place, after which a final phone call is made for assurance of stopping of medication and assessment of adverse events in the tapering period.

Dose reductions are accepted on the discretion of the principle investigator in case of dose-related side-effects (especially headaches) that would prevent the subject from continuing the study. In the case of rash, the patient should be taken off medication immediately.

**Dose escalation**				**Maintenance**	**Tapering**
Weeks 1+2	Weeks 3+4	Weeks 5+6	Weeks 7+8	9-26	Week 27+28
25 mg/d	50 mg/d	100 mg/d	150 mg/d	200 mg/d	100 mg/d
1 tablet of 25 mg (once daily)	2 tablets of 25 mg (once daily)	2 tablets of 25 mg (twice daily)	3 tablets of 25 mg (twice daily)	1 tablet of 100 mg (twice daily)	1 tablet of 100 mg (once daily)

7.6 Preparation and labeling of Investigational Medicinal Product

In section D is an example of the label for the lamotrigine or placebo containers. 25 mg/d tablets (for weeks 1 to 8) are provided in one container of 210 25 mg tablets (verum or placebo). 100 mg/d tablets are dispensed in two containers of 161 tablets (for weeks 9 to 18, and 19 to 29).

7.7 Drug accountability

The pharmacy of Erasmus MC (contact: dr. R. Bouamar; qualified person for batch release: prof.dr. Vulto) will take responsibility of disposal and accountability of returned study medication.

8. METHODS

8.1 Study parameters/endpoints

8.1.1 Main study parameter/endpoint

Cognitive functioning

- Performal intelligence (Wechsler Scales for Intelligence: 12 – 16 yrs: WISC-III-NL; 17-18 yrs: WAIS).

8.1.2 Secondary study parameters/endpoints

Neuropsychological tests (60 minutes)

Visual spatial learning efficacy

- Paired Associate Learning (from the CANTAB, Cambridge Neuropsychological Test Automated Battery)

Visual perception

- Motor-free Visual Perception Test – 3 (MVPT)

Attention

- Sustained Attention Dots (SADOTS, from the ANT, Amsterdam Neuropsychological Tasks)

Fine motor coordination

- Grooved Pegboard Test
- Beery Visual Motor Integration/ motor coordination (Beery-VMI-6)

Parent-questionnaires (25 minutes)

Attention problems

- Parent reported ADHD-questionnaire (AVL, ADHD Vragenlijst)

Executive functioning

- Behavior Rating Inventory of Executive function (BRIEF- questionnaire)

Neurophysiology (With TMS) (90 minutes)

Intracortical inhibition

- Short-interval Intracortical Inhibition (SICI), measured by paired pulse stimulation

Cortical plasticity

- LTP-like plasticity, measured by paired associative stimulation (PAS)

8.1.3 Process measurements

- Safety measures: ALAT/ASAT, gammaGT, ureum, creatinine, complete blood count.

- Lamotrigine blood levels: a through level, and levels at 3 and 6 hours post tablet intake.
- Clinical characteristics of participants (e.g. height, weight, blood pressure, disease severity, etc)
- Compliance to study medication (by counting returned tablets)
- Adverse event registration.

8.2 Randomisation, blinding and treatment allocation

Computer-generated permuted-block randomisation lists (block size of 6) will be generated by the department of biostatistics, Erasmus MC. Separate lists are generated for each participating centre. The pharmacist at Erasmus MC will receive the list starting with numbers LN001 to LN060, the pharmacist at UZ Leuven will receive a list starting with LN101 to LN160, the pharmacist at KBO-Kinderzentrum München/Technische Universität München will receive a list starting with LN201 to LN260, the pharmacist at University of Manchester will receive a list starting with LN301 to LN3060 and the pharmacist at University of Padova will receive a list starting with LN401 to LN460. The pharmacist at Hospital University of Sant Joan de Deu will receive a list starting with LN501 to LN560. Only the pharmacists have access to the randomisation lists. When a participant is included in the study by the site investigator, the study number will be issued by the pharmacist in the order of enrolment in the trial. All investigators, outcome assessors, parents, participants and data analysts will be blinded for treatment allocation, until the database is locked. To guarantee blinding, investigators are not allowed to open the medication boxes of the participants.

8.3 Study procedures

Procedures per visit are listed below:

T = - 1 week: Inclusion visit A:

- Assessment of inclusion/exclusion criteria
- Safety checklist (suicide-risk checklist, checklist for transcranial magnetic stimulation)
- Pregnancy testing in female participants is performed by dipstick testing in urine
- Blood finger prick: ALAT/ASAT, gammaGT, ureum, creatinine, complete blood count
- Neuropsychological tests. All neuropsychological tests described above are developed for children, and will be administered in Dutch versions. Before- and after testing will be administered by the same neuropsychologist. Questionnaires will be filled out by the same caregiver before and after.

T = 0 weeks: Inclusion visit B:

EMG and TMS measurements: the measurements described below are based on the procedures as used within the TMS lab of prof. V. Mall (Freiburg / München). Participants will only be allowed to be subjected to TMS measurements when no contraindications are present according to internationally safety guidelines [15]. These include: frequent occurrence of fainting spell or syncope, severe (i.e., followed by loss of consciousness) head trauma, pregnancy, metal in the brain/skull (except titanium), cochlear implants, implanted neurostimulator, cardiac pacemaker, surgical procedures to spinal cord/brain.

- Electromyographic (EMG) recording: motor evoked potentials (MEPs) are recorded from the left abductor pollicis brevis (APB) thumb muscle at rest by means of surface EMG, using silver/silver chloride electrodes in belly-tendon recording technique. Data are band-pass filtered (20-2000Hz) and amplified using a universal amplifier, digitized at 5 kHz and stored on a personal computer for online visual display and later offline analysis using Signal Software version 5 (CED Ltd., UK).

- Transcranial magnetic stimulation: a figure of eight shaped stimulation coil (MC-B70) is connected to a MagPro TMS stimulator (MagPro X100 with MagOption; MagVenture, Denmark) and centered tangentially on the scalp over the right primary motor cortex (M1) with its handle pointing in a posterior direction and laterally at an angle of approximately 45° away from the midline. The optimal position for eliciting MEPs of maximum amplitude from the left APB is determined first. In order to establish and maintain optimal coil positioning on the motor cortex, a 3D neuronavigation system is used (Visor2 XT). Resting motor threshold (rMT) is subsequently determined using a maximum likelihood threshold-hunting procedure (Croarkin et al., 2012). We will use 16 TMS stimuli starting at 45% of maximum stimulus output. A positive MEP is defined as a muscle activation of > 50 µV (CMAP amplitude). The initial stimulator output is chosen to target a mean MEP-amplitude of 800-1200 µV (SI1mV) and is then kept constant throughout the investigation to assess changes in MEPs. MEP size is determined by measuring the two highest peaks of opposite polarity and then averaged over 20 trials for each point of investigation.

- Paired pulse TMS (ppTMS): ppTMS is capable of evaluating intracortical inhibition by using different inter-pulse intervals to evaluate excitatory and inhibitory mechanisms. Short-interval intracortical inhibition (SICI) is measured according to the technique of

Kujirai et al. (Kijurai et al., 1993). For SICI, a conditioning stimulus with an intensity of 80% of rMT precedes a test stimulus with SI1mV. The interstimulus intervals will be 3 ms (SICI). To target a baseline inhibition of approximately 50%, we will additionally record SICI with 60% of rMT conditioning stimulus intensity. We will record 10 trials for each paired pulse condition of each level of subthreshold stimulation (60%and 80%). In addition, 10 single test stimuli will be given, thus at SI1mV intensity. This will result in a total number of 20 paired and 10 single pulses. These 3 conditions of paired or single pulse stimulations will be delivered in a randomized order. The intertrial interval will vary between 4-6 s to avoid habituation with repeated stimulation. Intracortical inhibition is as the ratio of conditioned to baseline MEP for each ISI. Thus, ppTMS provides insights into cortical excitation/inhibition balance.

- Paired associative stimulation and TMS (PAS TMS): PAS consists of 200 paired stimuli at a frequency of 0.25 Hz. Peripheral electrical stimulation of the median nerve at the wrist of the left hand is followed by TMS of the right hemispheric M1 at the same optimal site to elicit MEPs in the left APB muscle. Electrical stimulation will be applied through a stimulator using a bipolar electrode with the cathode proximal. We first identify the optimal stimulation site at the wrist, affix the electrode, and determine the sensory perception threshold (PT). During PAS, constant current square wave pulses with duration of 1000 µs are applied at an intensity of 3 times the PT. TMS intensity is set to elicit a mean MEP peak-to-peak amplitude of, on average, 1 mV, determined before the PAS procedure. This intensity is used throughout the experiment for post-PAS evaluation. The interstimulus interval between electrical and transcranial magnetic stimulation is 25 ms (PAS_{25}). Since the subject's level of attention may influence the direction and magnitude of the PAS_{25} effect, we will repeatedly remind subjects to focus their attention on the stimulated hand and to count the number of electrical stimuli. The PAS effect is determined on the mean of all time points after PAS compared to baseline. Mean MEP amplitude is measured before PAS (pre-PAS), and at 3 time points after the PAS TMS procedure (0 min; 30 min; 60 min), each time based on the average of 20 trials.

T=4 weeks: Telephone call to monitor adverse events and compliance. Study guidance.

T=8 weeks: Telephone call to monitor adverse events and compliance. Study guidance.

T=10 weeks: Hospital visit: TMS measurements as in inclusion visit B plus ADHD questionnaire, neurological exam, venipuncture to obtain samples for safety parameters

(ASAT/ALAT, gammaGT, complete blood count) and lamotrigine levels at 3 hours post administration.

Preceding this appointment, parents and participants will be asked whether they agree on drawing extra blood (2x10mL) at this time point for the ENCORE Biobank study (MEC-2012-318). A separate informed consent procedure will be carried out. If participants agree, this will not result in an extra venipuncture. Extra blood will be drawn during the already planned venipuncture in this study.

T=14 weeks: Telephone call to monitor adverse events and compliance. Study guidance.

T=18 weeks: home visit, assessing adverse events and compliance, handing out medication, lamotrigine blood level (finger prick) at either t=6 hours post dose or just prior to the next dose.

T=26 weeks: Outcome-visit A: questionnaires and neuropsychological test, same as inclusion visit A. lamotrigine blood level (finger prick) at either t=6 hours post dose or just prior to the next dose.

T=28 weeks: Telephone contact, end of study.

T=52 weeks: Postal ADHD- and BRIEF- questionnaire, telephone contact about post-study period.

A graphical representation of all study procedures is provided on the next page.

Measure	T=-1	T=0	T=4	T=8	T=10	T=14	T=18	T=22	T=26	T=28	T=52
	Visit - Hospital	Visit - Hospital	☎	☎	Visit - Hospital	☎	Home visit	☎	Visit – Hospital	☎	☎
**Outcome**											
WISC-IIINL – performal IQ	■								■		
CANTAB – PAL	■								■		
MVPT	■								■		
SA-DOTS	■								■		
Grooved Pegboard	■								■		
VMI-6	■								■		
ADHD-questionnaire	■				■				■		■
BRIEF-questionnaire	■								■		■
TMS – SICI		■			■						
TMS – PAS		■			■						
**Safety measures**											
ALAT/ASAT/gammaGT	■				■						
Ureum, creatine	■										
Pregnancy test	■										
Lamotrigine levels					■		■		■		
Blood count	■				■						
Neurological examination	■				■				■		
History	■		■	■	■	■	■	■	■	■	■
Counting tablets									■		

8.4 Withdrawal of individual subjects

Subjects can leave the study at any time for any reason if they wish to do so without any consequences for clinical care. The investigator can decide to withdraw a subject from the study for urgent medical reasons. Individuals will be withdrawn from study medication in case of skin rash. Subjects are offered and encouraged to participate in the outcome assessment, in order to uphold the intention to treat principle.

8.5 Follow-up of subjects withdrawn from treatment

Individuals that have been withdrawn from treatment based on skin rash are considered to have had an allergic reaction and should never use lamotrigine again. All subjects are in follow-up at the ENCORE outpatient clinic and continue to be so after withdrawal from treatment. When treatment is stopped for reasons other than an emergency, a dose reduction phase of two weeks should be implemented.

8.6 Premature termination of the study

The steering committee can decide on the premature termination of the study. The DSMB can advise at any moment to the steering committee that the study should be terminated or whether the safety regulations of the study should be adjusted based on the following safety analyses:

1. SAE's will be reported to the DSMB within 7 days. The DSMB will receive this information blinded in first instance. In case of a grade 4 or 5 adverse event, which is reasonably related to the use of lamotrigine or otherwise related to the nature of the study, they may choose to analyze this patient unblinded.
2. Interim analysis for safety (by the DSMB) will be performed when asked for by principle investigator and on a fixed basis after 20 and 40 subjects have completed the study. For such an analysis, they will be provided with a coded list of included patients (including lost to follow-up numbers), recent relevant literature and a list of all adverse events reported by participants. In case, adverse events grade 2 and 3 seem to occur more often than can be expected based on the information in the SPC, they can choose to analyze these patients unblinded.

The study will be terminated if at an interim analysis it appears that continuing the study is futile. Futility, in this case, is defined as a drop out rate of 50% of included patients (with a minimum of 20 patients included) by T=10 due to side effects.

No interim analysis will be performed on positive efficacy as in this small proof-of-principle study, effect sizes should be very robust at these time points to proof efficacy. We consider this chance to be fairly small, and not outweighing the consequences of multiple testing.

To enable intention-to-treat analysis, neither the investigator, nor the participant will be informed on the result of unblinding by the DSMB. This also holds for situations in which participants leave the study as of SAE's.

9. SAFETY REPORTING

9.1 Section 10 WMO event

In accordance to section 10, subsection 1, of the WMO, the investigator will inform the subjects and the reviewing accredited METC if anything occurs, on the basis of which it appears that the disadvantages of participation may be significantly greater than was foreseen in the research proposal. The study will be suspended pending further review by the accredited METC, except insofar as suspension would jeopardise the subjects' health. The investigator will take care that all subjects are kept informed.

9.2 Adverse and serious adverse events

Adverse events are defined as any undesirable experience occurring to a subject during the study, whether or not considered related to lamotrigine. All adverse events reported spontaneously by the subject or observed by the investigator or his staff will be recorded.

A serious adverse event is any untoward medical occurrence or effect that at any dose:

- results in death;
- is life threatening (at the time of the event);
- requires hospitalisation or prolongation of existing inpatients' hospitalisation;
- results in persistent or significant disability or incapacity;
- is a congenital anomaly or birth defect;
- is a new event of the trial likely to affect the safety of the subjects, such as an unexpected outcome of an adverse reaction, lack of efficacy of an IMP used for the treatment of a life threatening disease, major safety finding from a newly completed animal study, etc.

All SAEs will be reported through the web portal *ToetsingOnline* to the accredited METC that approved the protocol, within 15 days after the sponsor has first knowledge of the serious adverse reactions.

SAEs that result in death or are life threatening should be reported expedited. The expedited reporting will occur not later than 7 days after the responsible investigator has first knowledge of the adverse reaction. This is for a preliminary report with another 8 days for completion of the report.

9.2.1 Suspected unexpected serious adverse reactions (SUSAR)

Adverse reactions are all untoward and unintended responses to an investigational product related to any dose administered.

Unexpected adverse reactions are adverse reactions, of which the nature, or severity, is not consistent with the applicable product information (e.g. Investigator's Brochure for an unapproved IMP or Summary of Product Characteristics (SPC) for an authorised medicinal product).

If a SUSAR is suspected, the investigator will notify the DSMB, and requests an expedited advise. The advice may include to break the code for this patient.

The sponsor will report expedited the following SUSARs through the web portal *ToetsingOnline* to the METC:

- SUSARs that have arisen in the clinical trial that was assessed by the METC;
- SUSARs that have arisen in other clinical trials of the same sponsor and with the same medicinal product, and that could have consequences for the safety of the subjects involved in the clinical trial that was assessed by the METC.

The remaining SUSARs are recorded in an overview list (line-listing) that will be submitted once every half year to the METC. This line-listing provides an overview of all SUSARs from the study medicine, accompanied by a brief report highlighting the main points of concern.

The expedited reporting of SUSARs through the web portal ToetsingOnline is sufficient as notification to the competent authority.

The sponsor will report expedited all SUSARs to the competent authorities in other Member States, according to the requirements of the Member States.

The expedited reporting will occur not later than 15 days after the sponsor has first knowledge of the adverse reactions. For fatal or life threatening cases the term will be maximal 7 days for a preliminary report with another 8 days for completion of the report.

9.2.2 Annual safety report

In addition to the expedited reporting of SUSARs, the sponsor will submit, once a year throughout the clinical trial, a safety report to the accredited METC, competent authority, Medicine Evaluation Board and competent authorities of the concerned Member States.

This safety report consists of:

- a list of all suspected (unexpected or expected) serious adverse reactions, along with an aggregated summary table of all reported serious adverse reactions, ordered by organ system, per study;
- a report concerning the safety of the subjects, consisting of a complete safety analysis and an evaluation of the balance between the efficacy and the harmfulness of the medicine under investigation.

9.3 Follow-up of adverse events

All adverse events will be followed until they have abated, or until a stable situation has been reached. Depending on the event, follow up may require additional tests or medical procedures as indicated, and/or referral to the general physician or a medical specialist.

9.4 Data Safety Monitoring Board (DSMB)

DSMB will consist of persons independent from the research group and have acknowledged to have no conflict of interest.

Dr. R.F. Neuteboom, neurologist, pediatric neurologist
Drs. J. Drenthen, neurologist, clinical neurophysiologist
Dr. C.R. Lincke, pediatrician
Prof.dr. E. Steyerberg (clinical epidemiologist, statistician)

The advice(s) of the DSMB will be notified upon receipt by the sponsor to the METC that approved the protocol. With this notification a statement will be included indicating whether the advice will be followed. The operating principles of the DSMB are specified in paragraph 7.6.

10. STATISTICAL ANALYSIS

Predefined primary analysis will be done according to the intention to treat principle. Differences on all primary and secondary outcome measures between the lamotrigine and placebo groups after 26 weeks of treatment will be assessed using bivariable (adjusted for baseline scores), and multivariable linear regression analysis adjusted for baseline scores, age and sex. The cut-off level for significance will be set at $p<0.05$. We will not adjust the alpha-level for multiple hypothesis testing in the secondary outcome measures. Adjustment would be problematic due to unknown interdependence of the outcome measurements and the associated rise in type II error rate, when corrected. The interpretation of these secondary outcome measures will be in agreement with their secondary nature. The purpose of the inclusion of the secondary outcome measures is the explanation of effects or the absence thereof on the primary outcome measure.

Predefined exploratory analysis will consist of:

- Effect modification of outcome parameters that are significantly different between the treatment- and placebo group after 26 weeks will be examined using following interaction terms:
  1) Between treatment and age, as brain plasticity is conceivably higher in younger children.
  2) Between treatment and methylphenidate use, as on itself methylphenidate might improve certain outcome measures (prior to the study).
  3) Between treatment and $C_{av,ss}$ /AUC of lamotrigine plasma concentration, as lower $C_{av,ss}$/AUC could lead to a less prominent improvement. In order to determine pharmacokinetic characteristics such as $C_{av,ss}$ and AUC, a pharmacokinetic model will be build using NONMEM analysis of collected lamotrigine blood levels.
  4) Between treatment and baseline test score, because of the large room for improvement in children with baseline impairments.

  If the interaction between treatment and baseline test scores is significant, subgroup analysis will be performed for groups of participants with scores < –1SD of norm population.
- Per protocol analysis of the neurophysiology data. The rationale for this analysis is the scientific question whether lamotrigine is capable of restoring neuronal plasticity in individuals with NF1, and therefore, this exploratory analysis will only include subjects that have been >80% compliant as assessed by returned capsules and were on study medication at the time of outcome assessment.

ETHICAL CONSIDERATIONS

10.1 Regulation statement

This study will be conducted according to the principles of the Declaration of Helsinki (version: October 2008, Seoul) and in accordance with the Medical Research involving Human Subjects Act (WMO).

10.2 Recruitment and consent

Recruitment of NF1 patients will take place through outpatient clinics, and potential participants will be informed about the study by their treating physician.

If the potential participant shows interest in the study, the investigator will contact him or her and will send the PIF. At least three days later, the investigator will make a phone call to answer questions about this study. If the participant agrees on joining this study, an appointment will be made for the first visit. Informed consent is given by the participant at this visit.

10.3 Benefits and risks assessment, group relatedness

The potential benefit for participants is in the therapeutic effect of lamotrigine on cognitive symptoms. In addition, this study provides deeper insight in therapy with lamotrigine and mechanism of treatment effects, hopefully resulting in therapeutic options in the future. The participants can further expect a full reimbursement of travel costs and a reward of two cinema tickets.

The main risk of this study lies in the 6 month use of lamotrigine, an anti-epileptic drug that can cause some side-effects. Serious side-effects are rare and when emergent, can and will be countered promptly and effectively. There are no risks associated with the application of single pulse transcranial magnetic stimulation.

Total time investment for participants will be 11 hours, not including travel time. In our opinion, the goal of this research merits the execution of this protocol.

There are several reasons why we perform this study in children/adolescents instead of adults. These reasons are similar to trials previously performed in this population (MEC-2005-281 and MEC-2009-086).

We expect the children's/adolescent's brain to have the highest ability to change (most 'plastic').

Issues related to cognitive and behavioural deficits are most prominent in children/adolescents.

- The potential benefit of this study would directly apply to the study population.

- NF1-children/adolescents have a very characteristic profile of problems with school performance, behaviour and cognition. In contrast, at adult age, these problems have accumulated to a mild, broad range of cognitive deficits that are harder to quantify (probably because of adapting alternative problem-solving strategies).
- From a practical point of view, we can only use some of the outcome measures (e.g. some of the attention tasks, parent-rated questionnaires) by testing children/adolescents.
- Again from a practical point of view, there are no specific outpatient clinics for adult NF1 patients and the patients that are available often suffer from complex somatic complications of NF1, reducing generalizability of the results of this trial. In contrast, the outpatient clinics of the Sophia's Children's Hospital, UZ Leuven and KBO-Kinderzentrum München/Technische Universität Münchenare representative of children with NF1 because the clinics are easily accessible and children remain in yearly follow-up after diagnosis.
- Finally, the lower limit of 12 years is chosen, as dosages under this age limit are calculated according to body weight (kilograms) and will result in a large number of different dosages. In this proof-of principle study it is not feasible to produce placebo's for every possible dosage.

10.4 Compensation for injury

The sponsor/investigator has a liability insurance which is in accordance with article 7, subsection 6 of the WMO.

The sponsor (also) has an insurance which is in accordance with the legal requirements in the Netherlands (Article 7 WMO and the Measure regarding Compulsory Insurance for Clinical Research in Humans of 23th June 2003). This insurance provides cover for damage to research subjects through injury or death caused by the study.

1. € 450.000,-- (i.e. four hundred and fifty thousand Euro) for death or injury for each subject who participates in the Research;
2. € 3.500.000,-- (i.e. three million five hundred thousand Euro) for death or injury for all subjects who participate in the Research;
3. € 5.000.000,-- (i.e. five million Euro) for the total damage incurred by the organisation for all damage disclosed by scientific research for the Sponsor as 'verrichter' in the meaning of said Act in each year of insurance coverage.

The insurance applies to the damage that becomes apparent during the study or within 4 years after the end of the study.

10.5 Incentives

The Dutch participants will receive restitution of travel costs and two cinema tickets. This is independent of completion of the study.

11. ADMINISTRATIVE ASPECTS AND PUBLICATION

11.1 Handling and storage of data and documents

All data will be coded with LNxxx, where L is LTG and N is Neurofibromatosis. Xx represents the follow-up number of inclusion. Subjects at Erasmus MC will receive code 001 to 060, at UZ Leuven 101 to 160, in Munich 201 to 260, in Manchester 301 to 360, in Padova 401 to 460 and in Barcelona 501 to 560. In all reports, study documentation and publications, this code will be used. All participant-related information will be stored in a clinical report form, which will cover all outcome data, concomitant medication, consult information, therapy adherence etcetera. Relevant clinical data will be added to the clinical status of the participant by the treating physician. The clinical status of the participant will also be marked during the study to show participation in this trial. The treating physician and trial pharmacist will have the key to the subject identification code. Data will be stored for 15 years. The handling of personal data will comply with the Dutch Personal Data Protection Act (WBP).

11.2 Amendments

A ‘substantial amendment’ is defined as an amendment to the terms of the METC application, or to the protocol or any other supporting documentation, that is likely to affect to a significant degree:

- the safety or physical or mental integrity of the subjects of the trial;
- the scientific value of the trial;
- the conduct or management of the trial; or
- the quality or safety of any intervention used in the trial.

All substantial amendments will be notified to the METC and to the competent authority.

Non-substantial amendments will not be notified to the accredited METC and the competent authority, but will be recorded and filed by the sponsor.

11.3 Annual progress report

The sponsor/investigator will submit a summary of the progress of the trial to the accredited METC once a year. Information will be provided on the date of inclusion of the first subject, numbers of subjects included and numbers of subjects that have completed the trial, serious adverse events/ serious adverse reactions, other problems, and amendments.

11.4 End of study report

The sponsor will notify the accredited METC and the competent authority of the end of the study within a period of 90 days. The end of the study is defined as the last patient's telephone call at T=28.

In case the study is ended prematurely, the sponsor will notify the accredited METC and the competent authority within 15 days, including the reasons for the premature termination.

Within one year after the end of the study, the investigator/sponsor will submit a final study report with the results of the study, including any publications/abstracts of the study, to the accredited METC and the Competent Authority.

11.5 Public disclosure and publication policy

All results of this study will be disclosed unreservedly.

12. REFERENCES

1. Krab, L.C., et al., *Impact of neurofibromatosis type 1 on school performance.* J Child Neurol, 2008. **23**(9): p. 1002-10.
2. Graf, A., et al., *Quality of life and psychological adjustment in children and adolescents with neurofibromatosis type 1.* J Pediatr, 2006. **149**(3): p. 348-53.
3. Krab, L.C., et al., *Health-Related Quality Of Life in Children with Neurofibromatosis Type 1: Contribution of Demographic Factors, Disease-Related Factors, and Behavior.* J Pediatr, 2008.
4. Hyman, S.L., A. Shores, and K.N. North, *The nature and frequency of cognitive deficits in children with neurofibromatosis type 1.* Neurology, 2005. **65**(7): p. 1037-44.
5. Krab, L.C., et al., *Effect of simvastatin on cognitive functioning in children with neurofibromatosis type 1: a randomized controlled trial.* JAMA, 2008. **300**(3): p. 287-94.
6. van der Vaart, T., et al., *Simvastatin for cognitive deficits and behavioural problems in patients with neurofibromatosis type 1 (NF1-SIMCODA): a randomised, placebo-controlled trial.* Lancet Neurol, 2013. **12**(11): p. 1076-83.
7. Costa, R.M., et al., *Mechanism for the learning deficits in a mouse model of neurofibromatosis type 1.* Nature, 2002. **415**(6871): p. 526-30.
8. Cui, Y., et al., *Neurofibromin regulation of ERK signaling modulates GABA release and learning.* Cell, 2008. **135**(3): p. 549-60.
9. Shilyansky, C., et al., *Neurofibromin regulates corticostriatal inhibitory networks during working memory performance.* Proc Natl Acad Sci U S A, 2010. **107**(29): p. 13141-6.
10. Shilyansky, C., Y.S. Lee, and A.J. Silva, *Molecular and cellular mechanisms of learning disabilities: a focus on NF1.* Annu Rev Neurosci, 2010. **33**: p. 221-43.
11. Poolos, N.P., M. Migliore, and D. Johnston, *Pharmacological upregulation of h-channels reduces the excitability of pyramidal neuron dendrites.* Nat Neurosci, 2002. **5**(8): p. 767-74.
12. Barron, T.F., et al., *Lamotrigine monotherapy in children.* Pediatr Neurol, 2000. **23**(2): p. 160-3.
13. Neitz, A., et al., *Postsynaptic NO/cGMP Increases NMDA Receptor Currents via Hyperpolarization-Activated Cyclic Nucleotide-Gated Channels in the Hippocampus.* Cereb Cortex, 2013.
14. Delvendahl, I., et al., *Effects of lamotrigine on human motor cortex plasticity.* Clin Neurophysiol, 2013. **124**(1): p. 148-53.
15. Rossi, S., et al., *Safety, ethical considerations, and application guidelines for the use of transcranial magnetic stimulation in clinical practice and research.* Clin Neurophysiol, 2009. **120**(12): p. 2008-39.
16. Garvey, M.A. and V. Mall, *Transcranial magnetic stimulation in children.* Clin Neurophysiol, 2008. **119**(5): p. 973-84.
17. Mainberger, F., et al., *Lovastatin improves impaired synaptic plasticity and phasic alertness in patients with neurofibromatosis type 1.* BMC Neurol, 2013. **13**(1): p. 131.
18. Mainberger, F., et al., *Impaired motor cortex plasticity in patients with Noonan syndrome.* Clin Neurophysiol, 2013.
19. Jung, N.H., et al., *Impaired induction of long-term potentiation-like plasticity in patients with high-functioning autism and Asperger syndrome.* Dev Med Child Neurol, 2013. **55**(1): p. 83-9.
20. Gilbert, D.L., et al., *Should transcranial magnetic stimulation research in children be considered minimal risk?* Clin Neurophysiol, 2004. **115**(8): p. 1730-9.
21. U.S. Food and Drug Administration. *Safety Alerts for Human Medicinal Products. Antiepileptic Drugs*. 2008; Available from: http://www.fda.gov/Safety/MedWatch/SafetyInformation/SafetyAlertsforHumanMedicalProducts/ucm074939.htm.
22. European Medicines Agency. *Meeting highlights from the Committee for Medicinal Products for Human Use, 15-18 December 2008*. 2008; Available from: http://www.ema.europa.eu/ema/index.jsp?curl=pages/news_and_events/news/2009/11/news_detail_000226.jsp&mid=WC0b01ac058004d5c1.

23. Reagan-Shaw, S., M. Nihal, and N. Ahmad, *Dose translation from animal to human studies revisited.* FASEB J, 2008. **22**(3): p. 659-61.
24. U.S. Food and Drug Administration. *Guidance for Industry Estimating the Maximum Safe Starting Dose in Initial Clinical Trials for Therapeutics in Adult Healthy Volunteers.* 2005; Available from: http://www.fda.gov/downloads/Drugs/Guidances/UCM078932.pdf.
25. Evans, D. G. R., Salvador, H., Chang, V. Y., Erez, A., Voss, S. D., Schneider, K. W., ... & Tabori, U. (2017). Cancer and central nervous system tumor surveillance in pediatric neurofibromatosis 1. *Clinical Cancer Research*, *23*(12), e46-e53.
