# Supplementary material for: Lamotrigine for cognitive deficits associated with neurofibromatosis type 1: A phase II randomized placebo‐controlled trial
Source: Dev Med Child Neurol. 2024 Sep 28;67(4):537–49. doi: 10.1111/dmcn.16094 (PMC11875526; doi:10.1111/dmcn.16094)
Supplement: Supplementary file 2 — Appendix S2: Statistical analysis plan. [file DMCN-67-537-s004.pdf]

The effect of lamotrigine on cognitive deficits associated with Neurofibromatosis type 1: a phase II randomized, placebo-controlled, multi-centre trial (NF1-EXCEL)

Statistical Analysis Plan

Version 1, March 2021

Table of Contents

1. Administrative information 3
1.1 Study Title 3
1.2 Trial registration 3
1.3 Inclusion sites 3
1.4 Latest IRB protocol version 3
1.5 Statistical analysis plan version 3
1.6 IRB protocol and Statistical analysis plan version history 3
1.7 Contributors statistical analysis plan 4
1.8 Signatures 5

2. Introduction 6
2.1 Background and rationale 6
2.2 Objectives 6

3. Study Methods 7
3.1 Trial design 7
3.2 Randomization 7
3.3 Sample size calculation 7
3.4 Hypothesis testing framework 7
3.5 Interim analyses 7
3.6 Guidelines for early stop 7
3.7 Premature ending of the NF1-EXCEL study 7
3.8 Timing of final analysis 8
3.9 Timing of outcome assessment 8

4. Statistical Principles 10
4.1 Confidence intervals and P-values 10
4.2 Analysis population 10
4.3 Intervention adherence 10
4.4 Protocol deviations 10
4.5 Important note on blinding 11

5. Trial Population 12
5.1 Screening data 12
5.2 Eligibility 12
5.3 Recruitment, inclusion and follow-up data 12
5.4 Baseline characteristics 13

6. Analysis 14
6.1 Detailed outcome definition 14
6.2 Preparation of analyses 16
6.3 Primary efficacy analysis 16
6.4 Presentation of treatment effects 17
6.5 Checking for model assumptions and remedial measures 17
6.6 Explorative analysis 17
6.7 Separate publication of Neurophysiological outcomes 18
6.8 Analysis of Harms 19

7. References 20

1. Administrative information

1.1 Study Title
"The effect of lamotrigine on cognitive deficits associated with Neurofibromatosis type 1: a phase II randomized, placebo-controlled, multi-centre trial (NF1-EXCEL)"

1.2 Trial registration
Trial is registered at www.clinicaltrials.gov : https://clinicaltrials.gov/ct2/show/NCT02256124

1.3 Inclusion sites
There are three inclusion sites for the NF1-EXCEL study:

- Erasmus MC (EMC) – departments of Pediatric Neurology, Pediatrics and Child and Adolescent Psychiatry and Psychology.
- UZ Leuven (UZL) – department of Human Genetics.
- Hospital Sant Joan de Déu (HSJD) – department of Neurocutaneous Diseases and Neuro-Oncology.

1.4 Latest IRB protocol version
MEC-2013-460 version 9, December 7, 2018 (EMC) (available online through the clinicaltrials.gov registration page, see section 1.2).

1.5 Statistical analysis plan version
Version 1, March 2021

1.6 IRB protocol and Statistical analysis plan version history

Version	Date	Timing	Specification of changes
IRB protocol version 3	November 2013	Before start of inclusion.	First approved version by the IRB. Definition of study rationale, study methods, statistical principles and statistical analyses before start of inclusion. These sections have not changed in later versions of the IRB protocol.
IRB protocol version 4	January 2015	During inclusion.	Improved description of time investment for study visits containing TMS measurements.
IRB protocol version 5	May 2015	During inclusion.	Addition of blood hemoglobin measurement at the inclusion visit (T-1) as an additional safety measure.
IRB protocol version 6	September 2015	During inclusion.	Update to enable the University of Manchester and the University of Padova as additional

			inclusion centers. Due to logistic reasons, these centers were not able to start inclusion.
IRB protocol version 7	February 2016	During inclusion.	Update to specify that participant travel reimbursement is center specific and according to local laws.
IRB protocol version 8	May 2018	During inclusion.	Update to enable the Hospital Sant Joan Déu (HSJD; Barcelona, Spain) to enter the study as an inclusion center. Additionally, the inclusion criteria for this center are adjusted. As at the HSJD the NF1 diagnosis is primarily determined based on clinical criteria. It was decided (and approved), that participants at the HSJD could also be included based on clinical criteria only, as long as the clinical diagnosis was not pigmentation criteria only (café-au-lait spots and freckling). In such a case, clinical diagnosis did have to be supplemented with a genetic diagnosis.
IRB protocol version 9	December 2018	Last version update before termination of study.	Last version of IRB protocol: no changes to study methods, statistical principles and statistical analyses as compared to version 3 (pre-inclusion) of the IRB protocol.<br>Update to indicate a change of supplier for the Lamotrigine and placebo tablets. The original supplier stopped producing Lamotrigine. The new supplier provided identical Lamotrigine tablets to the first supplier. The placebo tablets were similar but not identical to the Lamotrigine tablets (slightly thicker, no inscription). It was, therefore, ensured in the protocol that the investigators were not allowed to open the medication containers of the participants, to protect blinding of the investigators.
SAP version 1	March, 2021	After termination of study, before unblinding.	More detailed description of the statistical principles and statistical analyses described in the IRB protocol. Any changes to the initial description in the IRB protocol are specifically indicated in this version of the SAP.

1.7 Contributors statistical analysis plan

Name	Function	Email	Abbr.
Myrthe Ottenhoff	Coordinator	m.ottenhoff@erasmusmc.nl	MO
André Rietman	Neuropsychologist	a.rietman@erasmusmc.nl	AR
Jesminne Castricum	Co-site-coordinator	j.castricum@erasmusmc.nl	JC

Sabine Mous	Neuropsychologist	s.mous@erasmusmc.nl	SM
Joke Tulen	Psychophysiologist	j.h.m.tulen@erasmusmc.nl	JT
Hongchao Qi	Senior statistician	h.qi@erasmusmc.nl	HQ
Marie-Claire de Wit	Supervising coordinator	m.c.y.dewit@erasmusmc.nl	MW
Henriette Moll	Principle investigator	h.a.moll@erasmusmc.nl	HM
Ype Elgersma	Principle investigator	y.elgersma@erasmusmc.nl	YE

1.8 Signatures

Name	Function	Signature	Date
Myrthe Ottenhoff	Statistical analysis plan author/ Coordinator		02-06-2021
Hongchao Qi	Senior Statistician	Hongchao Qi	08-11-2021
Marie-Claire de Wit	Supervising Coordinating Investigator		24-09-2021
Ype Elgersma	Principal Investigator		03-06-2021
Henriette Moll	Principal Investigator		14-10-2021

2. Introduction

In this section we will give a summary of the study rationale and objectives. Full details can be found in the IRB protocol.

2.1 Background and rationale

Neurofibromatosis type I (NF1; incidence 1:3000) is one of the most common monogenetic causes of cognitive disability. It is an autosomal dominant disorder, caused by mutations in the NF1 gene, and characterized by a wide variability of cutaneous manifestations, neurofibromas, and cognitive, social, motor and emotional problems. Despite the frequency of the disorder and the impact on daily life, there is currently no evidence- based treatment targeting the cognitive problems in NF1. The ENCORE-laboratory at Erasmus MC has recently shown that the cognitive deficits in *Nf1* mice are caused by attenuated function of HCN-channels (hyperpolarization-activated cyclic nucleotide-gated channels) in interneurons. In mice, lamotrigine (LTG), an HCN-sodium channel agonist, rescues the neuronal plasticity and learning deficits. lamotrigine is approved to treat epilepsy and bipolar disorder, and is frequently used in children with and without NF1 to treat epilepsy. We hypothesize that lamotrigine will improve neuronal plasticity in adolescents with NF1 and improve their cognitive functioning.

2.2 Objectives

The objective of this proposal is to find proof-of-principle for an effect of lamotrigine on cognitive functioning in adolescents with Neurofibromatosis type 1.

Secondary objectives are the evaluation of the safety and the effect of lamotrigine on subdomains of cognitive function, intra-cortical inhibition and LTP-like plasticity.

3. Study Methods

In this section we will give a summary of the study methods. Full details can be found in the IRB protocol referenced in section 1.4.

3.1 Trial design

Randomized, placebo-controlled, double-blind, parallel-group clinical trial.

3.2 Randomization

Patients were randomized to lamotrigine or placebo in a 1:1 ratio. Randomization is stratified per center. For each center a computer-generated permuted-block randomization list is generated (block size of 6 participants).

3.3 Sample size calculation

The sample size for this study was calculated to be 30 subjects per group, or 60 subjects in total. Please see the latest version of the IRB protocol referenced in section 1 for the full details of this calculation.

3.4 Hypothesis testing framework

Analyses will be performed within a superiority hypothesis testing framework.

3.5 Interim analyses

There are no interim analyses planned on any of the primary or secondary outcome measures, as prespecified in the IRB protocol. Only safety data (adverse events) and basic patient descriptives are summarized at regular intervals for reporting to the data safety monitoring board (DSMB). This reporting is done for participants as one group and not per treatment group, meaning that the DSMB is kept blind. Therefore, the interim analyses have no influence on the methods for final outcome analysis.

3.6 Guidelines for early stop

The guidelines for an early stop are given in full in the IRB protocol, specifically in section 8.6. In summary, the study will be terminated in case of a 50% drop-out rate from when at least 20 participants are included. Additionally, the data-safety monitoring board can advise to stop the study based on the frequency of adverse events observed during the trial.

3.7 Premature ending of the NF1-EXCEL study

On 30-Apr-2020 it was decided to prematurely stop the NF1-EXCEL study. The reasons for premature stop where a combination of the following factors.

First, inclusion at the centers participating at the start of inclusion was lower than expected (EMC and UZL), mainly due to a lower willingness to participate. We, therefore, started collaboration with Hospital Sant Joan de Déu Barcelona (Spain) in the second half of 2019, however the inclusion rate at this center was also lower than expected.

Second, the COVID-19 pandemic prohibited inclusion of participants. By the end of March 2020 the HSJD had included 5 participants that were in the middle of follow-up. Due to severe lock-down restrictions in Spain the follow-up of these 5 participants had to be stopped. Additionally, we decided to open the inclusion in the Erasmus MC site again at the beginning of this year (2020). Since the last screening in the beginning of 2017, ±55 new potential candidates reached the age of 12, and perhaps there were new patients willing to participate. Only 5 patients of these potential candidates said 'yes' to start this study at the end of March 2020. However, also in the Netherlands COVID-19 restrictions prohibited us to include participants, to perform assessments or to recruit new patients.

Third, there were severe logistical problems concerning the study medication. We were using generically produced lamotrigine by TEVA. By 2020 TEVA had stopped producing the original study medication. Additionally, the pause in inclusion due to COVID-19 would mean that the study medication in possession of the research team would expire. This was a problem because our placebo was made to specifically mimic the TEVA medication. To start up new placebo production was financially unfeasible for this investigator initiated study.

Taken together these factors together, we have decided to discontinue the study.

3.8 Timing of final analysis

All primary and secondary outcome analyses will be performed collectively after terminating the study. Before analyses the following steps should be completed in the following order:

1. Dataset is finalized including data quality check on primary and secondary outcomes and all data queries are resolved.
2. Statistical analysis syntax is written using blinded data set.
3. Statistical analysis syntax is reviewed and approved by the senior statistician.
4. Unblinding of the study.
5. Analysis of primary and secondary outcomes.

3.9 Timing of outcome assessment

The table below indicates the timing of each outcome assessment. We indicate study visit times relative to the start of the intervention in weeks, e.g. T-1 indicates the study visit 1 week before start of the intervention. The intervention of this trial lasted for 26 weeks.

Study visits windows are defined as maximum 1 week prior and 2 weeks after the original study visit week. For example, the T10 study visit could only occur in week T9, T10, T11 or T12.

Study visit:	T-1	T0	T10	T26	T52
**Lamotrigine intervention:**		200mg daily including initial 8 week dosage build-up phase			
**Outcomes**					
PIQ	♦			♦	
CANTAB-PAL	♦			♦	
MVPT	♦			♦	
SA-DOTS	♦			♦	
Grooved Pegboard	♦			♦	
VMI-6	♦			♦	
ADHD questionnaire	♦		♦	♦	♦
BRIEF questionnaire	♦			♦	♦
TMS - SICI		♦	♦		
TMS - PAS		♦	♦		

Table 1: Outcome assessment timing. *♦ = baseline measurement; ♦ = outcome measurement; ♦ = extra measurement, not included as outcome for efficacy analysis. ADHD questionnaire = attention deficit hyperactivity disorder DSMB-based questionnaire; BRIEF questionnaire = behavior rating inventory of executive function questionnaire; CANTAB-PAL = Cambridge neuropsychological test automated battery – paired associates learning task; MVPT = motor-free visual perception test; PIQ = performance intelligence quotient; SA-DOTS = sustained attention dots task; TMS-SICI = transcranial magnetic stimulation short-interval intracortical inhibition; TMS-PAS = transcranial magnetic stimulation – paired associative stimulation.*

4. Statistical Principles

4.1 Confidence intervals and P-values

We will report means and 2-sided 95% confidence intervals of all outcomes defined in section 6. As predefined in the IRB protocol, the cut-off level for significance will be set at $p<0.05$ and we will not adjust the alpha-level for multiple testing (see also section 6.3 for a citation of the IRB protocol). As stated in the IRB protocol, adjustment would be problematic due to unknown interdependence of the outcome measurements and the associated rise in type II error rate when corrected.

4.2 Analysis population

We will perform complete case analysis, with all subjects analyzed by randomized group irrespective of compliance. Strictly speaking, such an analysis is considered to be a per-protocol analysis, assuming that there will be a small proportion of missing follow-up outcome data. Complete case analysis implies no imputation of these missing data and no exploration of the effect of this missing data, which violates the intention-to-treat principle [1–4]. Thus, while in the IRB protocol we propose an intention-to-treat analysis, our analysis has instead to be defined as a per-protocol analysis, assuming the presence of missing follow-up outcome data.

4.3 Intervention adherence

Adherence is defined as being on study medication at the time of follow-up outcome assessment and a >80% compliance, as assessed by the percentage of consumed tablets out of the expected number of consumed tablets. The expected number of consumed tablets is precisely calculated by the exact follow-up time per participant.

Compliance will be reported as median percentage as well as the 25th and 75th quantile, per intervention group. Additionally, the number of adherent participants will be reported.

At each study visit, participants received study medication in a quantity sufficient to make it till the next in-person study visit. The quantities of tablets given out were always slightly above the number of tablets they were expected to consume in that period. Participants were aware of this surplus. All quantities of tablets given out and returned were recorded in the CRF together with the date at which this occurred, to enable compliance calculation as described above.

4.4 Protocol deviations

We will summarize the following plausible protocol deviations in a (supplementary) table:

- <u>Outcome assessment missed or out of window:</u>
    - Study visit occurred >1 week prior or >2 weeks past the week in which the study visit should have been planned. Thus if the original study visit week is week T26, the visit should not occur earlier than week T25 or later than week T28. We will report the number of out-of-window visits and in how many participants these occurred.

    - A specific baseline or outcome assessment was not performed within a study visit. We will specify the number of missing assessments per time point (both baseline and T20 for neurophysiological outcomes and T26 for neuropsychological outcomes).
- Participant was randomized but did not meet the entry criteria: we will report the number of participants and at which point follow-up was terminated.
- Participant needed a dosage alteration because of adverse events: we will report the number of participants needing dose alterations, the median dosage adjustment in mg, the median period dose alteration was needed.
- Participant was required to start contra-indicated medication (as stated in the exclusion criteria): we will report the number of participants needing contra-indicated medication and the result of this prescription for study follow-up.

4.5 Important note on blinding

Prof. Eric Legius (EL), site-coordinator of the UZL, had access to Lamotrigine blood levels of the participants included at the UZ Leuven before the primary analysis and unblinding of the study. Due to storage capacity problems at the UZL, it was not possible to store lamotrigine blood samples for analysis after unblinding there. EL, therefore, had no role in the follow-up of participants, nor in the design or execution of the analyses. No other member of the study group in neither of the inclusion centers had access to this data before unblinding.

5. Trial Population

5.1 Screening data

Screening data will not be analyzed.

5.2 Eligibility

Below a summary of the eligibility criteria, see fully detailed criteria in the IRB protocol.

Inclusion criteria:

- Genetically confirmed NF1 diagnosis. (For the HSJD inclusion center: clinical NF1 diagnosis according to the NIH NF1 criteria was also accepted [5], as long as the clinical diagnosis was not based on pigmentation criteria only).
- Age 12-17.5 years at inclusion.
- Oral and written informed consent by parents and assent from participants.

Exclusion criteria

- Segmental NF1.
- Severe hearing problems or deafness.
- Severe visual problems or blindness.
- Use of medication that is known to influence Lamotrigine blood levels.
- Previous use of Lamotrigine.
- Previous allergic reactions to anti-epileptic drugs
- Epilepsy or epilepsy in the past.
- Suicidal thoughts or behavior.
- Renal and/or liver insufficiency.
- Pregnancy.
- Brain tumor or other brain pathology potentially influencing outcome measures.

5.3 Recruitment, inclusion and follow-up data

We will report the recruitment and inclusion data as a flow-chart. This flow chart will contain the following information:

- The number of subjects screened for eligibility.
  - The number of excluded subjects and the basis of exclusion.
- The number of eligible subjects.
  - The number of subjects that declined participation and the main reason for decline.
- The number of randomly assigned participants.
- Number of participants allocated per treatment group.
  - Number of participants lost to follow-up including reason.
  - Number of non-compliant participants (defined as a intervention adherence of <80%).
  - The number of participants analyzed.

5.4 Baseline characteristics

The baseline data will be presented in Table 1 of the trial manuscript. This table will show summary data per treatment group of the following variables:

- Age (years) at inclusion.
- Sex (female/male), reported as number and percentage female.
- Baseline performance, verbal and total intelligence quotient.
- NF1 disease severity (Riccardi scale), reported as percentage per severity level (minimal/mild/moderate/severe).
- *NF1* variant type, reported as percentage per variant category (missense/frameshift/splicing/non-sense/small-in frame deletion/large intragene duplication or deletion/chromosomal microdeletion).
- NF1 inheritance, reported as percentage per inheritance category (familial/ de novo).
- Education type, reported as percentage per type (regular/remedial teaching/special schooling).
- ASD diagnosis (percentage).
- ADHD diagnosis (percentage).
- Socio-economic status (postal code based Z-score).
- Methylphenidate use, reported as the number and percentage of participants using methylphenidate.

Whether continuous baseline variables are reported as median (25th-75th percentile) or mean (SD) is based on the expectation of their distribution. In case continuous baseline variables show significant deviance from a normal distribution, they will be reported as median (25th-75th percentile) instead.

6. Analysis

6.1 Detailed outcome definition

Below we will give detailed description of the outcomes used for analysis. An important note on the neuropsychological and questionnaire outcomes: unless an outcome is defined as a raw score, all neuropsychological test scores and questionnaire outcomes are standardized scores that are calculated using norm group data provided by the publisher of the test/questionnaire. Neuropsychological standardized scores, and especially z-scores, are not based on the mean and standard deviation found in the study population.

All outcomes are measured at inclusion and after either the full intervention of 26 weeks (T26) or after the Lamotrigine dose-escalation phase at 10 weeks (T10) (see Table 1 for overview).

6.1.1 Primary outcome

The Performance Intelligence Quotient (T26): standard score of PIQ measured with the Wechsler Intelligence Scale for Children version 3 (WISC-III). Norms used are specific to the country of the inclusion center.

For those children who were older than 16;11 years at the first (T-1) or the second measurement (T26), we used a linear extrapolation of normative values to obtain age-appropriate standard scores for each subscale of the primary outcome measure (WISC-PIQ). This was done using the following 4-step method:

1. The WISC normative scores are provided for age groups of 4 months (e.g. 16;4-16;7 or 16;8-16;11). Per subtest, we looked up the normative scores of the four oldest age categories.
2. We calculated the average score change over these last four categories.
3. We then applied that average score increase to 4 month age categories till we reached the age category of the participant.
4. When this process was completed for all subtests, scores were added up and the corresponding standard score was deduced from the standard score conversion table form the WISC-III manual.

*Example: A participant of 17 years and 5 months old had a raw score of 81 on the Coding subtests. The corresponding normative scores for the oldest age categories in the WISC-III manual are: 13 (15;8-15;11 years); 12 (16;0-16;3 years); 12 (16;4-16;7 years) and 11 (16;8-16;11 years). The average score change is (1+0+1)/3 = 0.67. To reach the participant's age category, we had to add two categories: 17;0-17;3 and 17;4-17;7. Therefore, the participant's normative score for Coding would be 11-(2*0.67)=9.67, which was rounded to 10. When all normative subtest scores were added, the performance score was 33, corresponding to an PIQ standard score of 76.*

6.1.2 Secondary neuropsychological and behavioral outcomes

Visuo-spatial learning efficacy (T26): total errors z-score of the Paired Associates Learning task from the CANTAB Research Suite.

Visual perception (T26): total z-score on the Motor-free Visual Perception Test version 3 (MVPT-3).

Attention (T26): session variation z-score on the Sustained attention Dots task (SA-Dots) from the Amsterdam Neuropsychological Tasks (ANT) battery. This variable is called the “sser_sd” in the ANT software output.

Fine motor coordination (T26):

- Raw score of the total test time using the dominant hand on the Grooved Pegboard Test.
- Motor coordination subtest z-score of the Beery-Buktenica Developmental Test of Visual Motor Integration version 6 (Beery-VMI-6).

Attention problems (T26): total z-score of the Parent reported ADHD-questionnaire (Aandachtsvragenlijst (AVL); a DSM-based questionnaire for ADHD symptoms).

Executive functioning (T26): global executive composite t-score of the parent reported Behavior Rating Inventory of Executive function (BRIEF).

6.1.3 Secondary neurophysiological outcomes

The neurophysiological outcomes are measured using transcranial magnetic stimulation (TMS) paradigms. TMS is used to elicit brain activity by applying non-invasive magnetic pulses through the skull. In this study, the TMS paradigms applied utilize magnetic pulses over the motor cortex to elicit motor-evoked potentials (MEPs) in the thumb. These MEPs are measured with electromyography. For each paradigm multiple MEPs are measured per subject and study visit. Below, we explain how a single TMS outcome for each participant and study visit is calculated from these multiple MEP measurements.

Cortical inhibition (T10):

Cortical inhibition a TMS paradigm called Short-Interval Intracortical Inhibition (SICI). Each SICI session contains three conditions: control single stimulations, paired 60%RMT stimulations, paired 80%RMT stimulations. Within each of these conditions 10 MEPs are measured per study visit, thus a total of 30 MEPs.

For the cortical inhibition outcome, we arrive at a single outcome by focusing on the difference in MEP size (mV) between the “control” and the “60%RMT” condition. For each of those two conditions, the median MEP size per subject will be calculated. Subsequently the “control” median will be subtracted from the “60% RMT” median, resulting in the final measure.

Cortical plasticity (T10):

Cortical plasticity is measured with the TMS paradigm Paired Associative Stimulation (PAS). Each PAS session contains five conditions: "baseline", "PAS induction", "post 1", "post 2" and "post 3". Within each of these conditions 20 MEPs are collected, except for the PAS induction condition that collects 200 MEPs.

To arrive at a single cortical plasticity measure, we focus on the difference between the "baseline" and "post 1" condition MEP size (mV). A subject specific median for each condition will be calculated. Subsequently, the "baseline" median will be subtracted from the "post 1" median, resulting in the final measure.

6.2 Preparation of analyses

To enable a swift data analysis phase, it is necessary to extract a practice analysis set. This will be done before unblinding and, therefore, the treatment group variable will be simulated for this analysis.

Using the practice analysis dataset, a syntax for analysis will be written in RStudio (v 1.3.1093 or higher) running on R software (v 4.0.3 or higher) by MO. The final version of the software will be reported in the publication. Analyses will require the additional R packages emmeans and ggplot2. Syntax construction will be supervised by HQ.

Based on the practice analysis data and syntax, it will be decided whether the SAP needs to be adjusted. If so, this will lead to a new version number. All changes relative to the previous version of the SAP will be explicitly indicated.

6.3 Primary efficacy analysis

The outline for the statistical analysis was predefined in the IRB protocol before start of inclusion. This predefinition has not changed from the first approved version of the IRB protocol (version 3) up to and including the last version of the IRB protocol (version 9), as indicated in section 1.6. The description of the primary efficacy analysis in the IRB protocol is as follows:

> *"Predefined primary analysis will be done according to the intention to treat principle. Differences on all primary and secondary outcome measures between the lamotrigine and placebo groups after 26 weeks of treatment will be assessed using bivariable (adjusted for baseline scores), and multivariable linear regression analysis adjusted for baseline scores, age and sex. The cut-off level for significance will be set at p<0.05. We will not adjust the alpha-level for multiple hypothesis testing in the secondary outcome measures. Adjustment would be problematic due to unknown interdependence of the outcome measurements and the associated rise in type II error rate, when corrected. The interpretation of these secondary outcome measures will be in agreement with their secondary nature. The purpose of the inclusion of the secondary outcome measures is the explanation of effects or the absence thereof on the primary outcome measure."*

Below we will further specify this analysis, including some changes that have been decided on after terminating the study but before unblinding.

The treatment effects of lamotrigine on the efficacy variables (primary and secondary) are parameters of interest in the primary and secondary analysis. Given that the outcome variables are all continuous, multiple linear regression analysis will be utilized to obtain

treatment effect estimates. As mentioned in the IRB protocol, a model adjusting for the baseline outcome measurement will be fitted, which is given by:

$$y_{Fi} = \beta_0 + \beta_1 \times y_{Bi} + \beta_2 \times Group_i + \epsilon_i,$$

where y_{Fi} is the outcome measurement at follow-up for the ith subject, β_0 is the intercept, β_1 is the effect of baseline outcome y_{Bi} on the follow-up outcome, β_2 is the treatment effect, and ϵ_i is the error term.

In contrast to what is stated in the IRB protocol, the covariates sex and baseline age will only be adjusted in the multiple linear regression model if there exists imbalance for these covariates between the treatment group and the control group (t-test, $p < 0.05$). We chose for this alteration to the original statistical plan due to the small group size subsequent to the premature termination of the study, as performing the sex and age adjusted models could otherwise unnecessarily harm the statistical power of the efficacy models.

6.4 Presentation of treatment effects

We will present treatment effects in a table as the Group coefficient (β_1, section 6.3), in addition to their 95% confidence interval (95%CI), as well as the mean and SD of each follow-up outcome per group and the number of subjects that entered the analysis.

Additionally, we will visualize standardized treatment effects and their 95%CIs of the primary outcome and all secondary outcomes in a forest plot. For this plot, all outcomes will be converted to study population based z-scores with positive scores indicating improvement of the outcome (favoring treatment) before entering analysis.

6.5 Checking for model assumptions and remedial measures

The assumptions of the multiple linear regression model including the equal variance and normality will be checked with the graphical approach.

The equal variance assumption will be checked using the residual plots. If the assumption is violated, a robust standard error estimator will be used for the regression coefficients.

The normality assumption of the error term can also be checked with residual plots. If there exists nonnormality for error terms, square root- or log-transformation for the outcome can be used.

6.6 Explorative analysis

In contrast to what is stated in the IRB protocol, we will not perform exploratory analysis for the neuropsychological outcomes. Due to lack of power consequential to the premature study stop, the research team decided to not execute the predefined explorative analysis laid out in the IRB protocol for the neuropsychological and questionnaire data. This decision was made before finalizing the first version of the analysis plan, and thus before unblinding. Neurophysiological data will undergo exploratory analysis for hypothesis generating purposes, see section 6.7.

The IRB protocol (description has not changed since version 3, see section 1.6) stated the following on the explorative analyses.

> *"Predefined exploratory analysis will consist of:*
>
> - *Effect modification of outcome parameters that are significantly different between the treatment- and placebo group after 26 weeks will be examined using following interaction terms:*
>
>   1) *Between treatment and age, as brain plasticity is conceivably higher in younger children.*
>   2) *Between treatment and methylphenidate use, as on itself methylphenidate might improve certain outcome measures (prior to the study).*
>   3) *Between treatment and $C_{av,ss}$ /AUC of lamotrigine plasma concentration, as lower $C_{av,ss}$/AUC could lead to a less prominent improvement. In order to determine pharmacokinetic characteristics such as $C_{av,ss}$ and AUC, a pharmacokinetic model will be build using NONMEM analysis of collected lamotrigine blood levels.*
>   4) *Between treatment and baseline test score, because of the large room for improvement in children with baseline impairments.*
>
>   *If the interaction between treatment and baseline test scores is significant, subgroup analysis will be performed for groups of participants with scores < –1SD of norm population.*
>
> - *Per protocol analysis of the neurophysiology data. The rationale for this analysis is the scientific question whether lamotrigine is capable of restoring neuronal plasticity in individuals with NF1, and therefore, this exploratory analysis will only include subjects that have been >80% compliant as assessed by returned capsules and were on study medication at the time of outcome assessment."*

6.7 Separate publication of Neurophysiological outcomes

We will publish the TMS data separate from the neuropsychological and questionnaire data. Any conclusion on the efficacy of lamotrigine on the TMS outcomes will be based on the primary efficacy analysis described in section 6.3 using the TMS outcomes defined in section 6.1.3.

In contrast to the neurophysiological data, we will perform exploratory analyses on efficacy on the neurophysiological outcomes. The objective for executing exploratory analyses is the generation of new hypotheses.

These exploratory analysis will include (but are not limited to):

- Analysis of treatment effect with a multi-level linear model, such to eliminate the possible data loss created by calculating subject specific medians for the primary analysis.
- Per protocol analysis defined in IRB protocol (see section 6.6).
- Analysis of MEP size during the PAS induction phase, using a multi-level linear model.
- Exploration of correlations between (treatment effects on) the neurocognitive outcomes PIQ, CANTAB-PAL, ANT-SA-dots, Grooved Pegboard and (treatment effects on) all neurophysiological outcome.

In the publication concerning the neurophysiological outcomes, we will explicitly state the secondary nature of the neurophysiological outcomes, the exploratory nature of the analyses and that these analyses were designed after unblinding of the study.

Additionally, the title of the publication will also immediately indicate the exploratory nature of the analyses, and will state: "*Exploratory analysis of the effect of lamotrigine on cortical inhibition and plasticity in adolescents with Neurofibromatosis type 1: a phase II randomized, placebo-controlled, multi-centre trial (NF1-EXCEL)*."

6.8 Analysis of Harms

All adverse events reported by participants in this study will be summarized. Adverse event data is collected using the WHO Adverse Reaction Terminology. These data will be presented in a cross tabulation, with the rows indicating the system-organ class (SOC) and the columns indicating the Common Terminology Criteria for Adverse Events (CTCAE) severity grade (grade 1-5). Quantification will be the number of adverse events per SOC and CTCAE grade, and the number of participants who suffered from the adverse event. Examples of SOCs are "gastro-intestinal system disorders", "endocrine disorders", "respiratory system disorders", etc. In case the frequency of adverse events per grade is low, we will re-scale grades as: "grade 1", "grade 2-3" and "grade 4-5").

Additionally, we will report the number of participant needing dosage alteration because of adverse events, as described in section 4.3 and 4.4.

7. References

1. Gupta, S. K. Intention-to-treat concept: A review. *Perspect Clin Res* **2**, 109–112 (2011).
2. Hollis, S. & Campbell, F. What is meant by intention to treat analysis? Survey of published randomised controlled trials. *BMJ* **319**, 670–674 (1999).
3. Montori, V. M. & Guyatt, G. H. Intention-to-treat principle. *CMAJ* **165**, 1339–1341 (2001).
4. Shah, P. B. Intention-to-treat and per-protocol analysis. *CMAJ* **183**, 696 (2011).
5. Ferner, R. E. *et al.* Guidelines for the diagnosis and management of individuals with neurofibromatosis 1. *Journal of medical genetics* **44**, 81–8 (2007).
