# Supplementary material for: Lamotrigine for cognitive deficits associated with neurofibromatosis type 1: A phase II randomized placebo‐controlled trial
Source: Dev Med Child Neurol. 2024 Sep 28;67(4):537–49. doi: 10.1111/dmcn.16094 (PMC11875526; doi:10.1111/dmcn.16094)
Supplement: Supplementary file 3 — Figure S1: Trial profile. [file DMCN-67-537-s005.pdf]

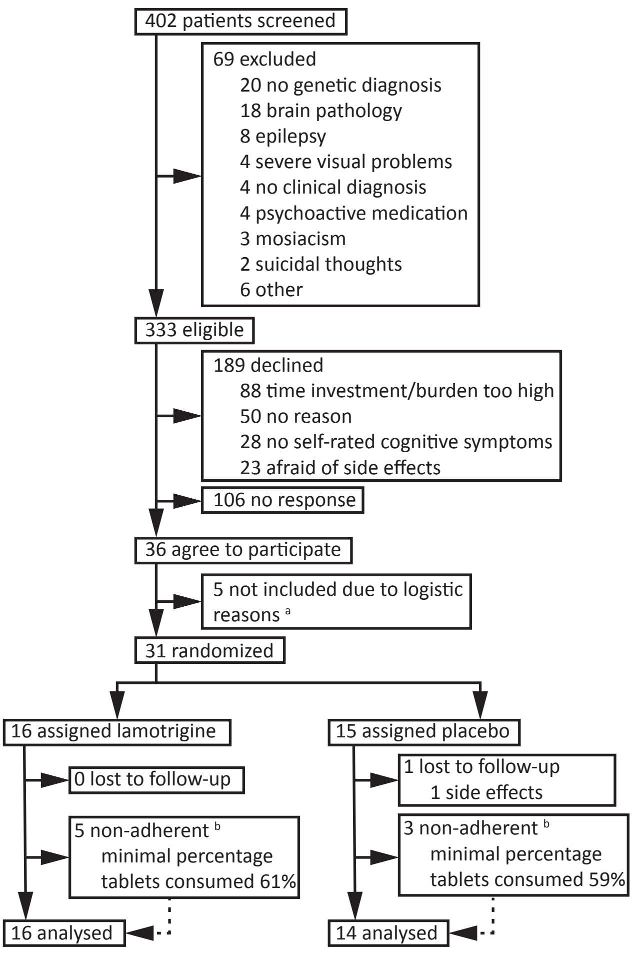
402 patients screened
69 excluded
20 no genetic diagnosis
18 brain pathology
8 epilepsy
4 severe visual problems
4 no clinical diagnosis
4 psychoactive medication
3 mosiacism
2 suicidal thoughts
6 other
333 eligible
189 declined
88 time investment/burden too high
50 no reason
28 no self-rated cognitive symptoms
23 afraid of side effects
106 no response
36 agree to participate
5 not included due to logistic reasons [a]
31 randomized
16 assigned lamotrigine
15 assigned placebo
0 lost to follow-up
1 lost to follow-up
1 side effects
5 non-adherent [b]
minimal percentage tablets consumed 61%
3 non-adherent [b]
minimal percentage tablets consumed 59%
16 analysed
14 analysed

FIGURE S1: Trial profile.

[a]Participants and parents had agreed to participate but were not able to enrol owing to COVID-19 restrictions and lack of resources.

[b]Adherence was defined as taking at least 80% of tablets over the course of the follow-up period.
