# Supplementary material for: Lamotrigine for cognitive deficits associated with neurofibromatosis type 1: A phase II randomized placebo‐controlled trial
Source: Dev Med Child Neurol. 2024 Sep 28;67(4):537–49. doi: 10.1111/dmcn.16094 (PMC11875526; doi:10.1111/dmcn.16094)
Supplement: Supplementary file 4 — Table S1: Alternative treatment effect models. [file DMCN-67-537-s002.docx]

**Table S1: Alternative treatment effect models.**

| Outcome | Model type | Treatment effect [95%CI] | *p*-value |
| --- | --- | --- | --- |
| CANTAB PAL Total errors z-score | linear model without RSE | 0.02 [-0.49, 0.54] | 0.93 |
| MVPT z-score | linear model without RSE | 0.4 [-0.27, 1.07] | 0.25 |
| ANT SA-Dots z-score | linear model without RSE | -0.81 [-1.66, 0.04] | 0.07 |
| Grooved Pegboard raw score | non-parametric | 3.72 [-1.58, 9.02]^a^ | 0.18 |
| AVL z-score | linear model on logarithmic transformed outcome | -0.03 [-0.43, 0.6]^b^ | 0.91 |
| BRIEF T-score | non-parametric | 0.65 [-3.4, 4.7]^a^ | 0.76 |

*Note:* RSE = robust standard error correction. ^a^ The unit of the treatment effect is a ranked score. ^b^ The treatment effect is obtained by applying the inverse logarithmic transformation, thus it is presented on the scale of the original score.
