# Supplementary material for: Lamotrigine for cognitive deficits associated with neurofibromatosis type 1: A phase II randomized placebo‐controlled trial
Source: Dev Med Child Neurol. 2024 Sep 28;67(4):537–49. doi: 10.1111/dmcn.16094 (PMC11875526; doi:10.1111/dmcn.16094)
Supplement: Supplementary file 5 — Table S2: Table of lamotrigine serum levels. [file DMCN-67-537-s001.docx]

**Table S2: Table of lamotrigine serum levels.**

|  | Tmax | Intermediate | Trough |
| --- | --- | --- | --- |
| Missing values, N (%) | 0 (0.00) | 1 (6.25) | 0 (0.00) |
| Lamotrigine (mg/L), median (range) | 5.96(1.93-12.77) | 5.05(0-8.76) | 4.68(1.54-7.36) |
| Therapeutic range, N (%) ^a^ | 15 (93.75) | 14 (93.33) | 15 (93.75) |
| Time after last intake (hrs), median (range) | 3.12 (1.67-5.75) | 5.72 (3.13-7.58) | 9.54 (6.8-11) |

*Note:* Summary of lamotrigine blood level analysis in the lamotrigine group (N = 16). Lamotrigine blood samples were drawn at 3 time points relative to the last lamotrigine tablet intake: a Tmax level aimed at around 3 hours post intake, and intermediate level aimed around 6 hours post intake and a trough level aimed as close to the evening dose intake as possible. ^a^ The therapeutic range was determined as 3-14 mg/L (35,36).
